# Genomic analysis of the TRIM family reveals two groups of genes with distinct evolutionary properties

**DOI:** 10.1186/1471-2148-8-225

**Published:** 2008-08-01

**Authors:** Marco Sardiello, Stefano Cairo, Bianca Fontanella, Andrea Ballabio, Germana Meroni

**Affiliations:** 1 Telethon Institute of Genetics and Medicine (TIGEM), Via P. Castellino 111, 80131 Naples, Italy; 2 Department of Pediatrics, Federico II University, Via Sergio Pansini 5, 80131 Naples, Italy; 3 Unite d'Oncogenèse et Virologie Moléculaire, Batiment Lwoff, Institut Pasteur, 28 rue de Dr. Roux, 75724 Paris Cedex 15, France; 4 Department of Pharmaceutical Sciences, University of Salerno, 84084 Fisciano (SA), Italy

## Abstract

**Background:**

The TRIM family is composed of multi-domain proteins that display the Tripartite Motif (RING, B-box and Coiled-coil) that can be associated with a C-terminal domain. TRIM genes are involved in ubiquitylation and are implicated in a variety of human pathologies, from Mendelian inherited disorders to cancer, and are also involved in cellular response to viral infection.

**Results:**

Here we defined the entire human TRIM family and also identified the TRIM sets of other vertebrate (mouse, rat, dog, cow, chicken, tetraodon, and zebrafish) and invertebrate species (fruitfly, worm, and ciona). By means of comparative analyses we found that, after assembly of the tripartite motif in an early metazoan ancestor, few types of C-terminal domains have been associated with this module during evolution and that an important increase in TRIM number occurred in vertebrate species concomitantly with the addition of the SPRY domain. We showed that the human TRIM family is split into two groups that differ in domain structure, genomic organization and evolutionary properties. Group 1 members present a variety of C-terminal domains, are highly conserved among vertebrate species, and are represented in invertebrates. Conversely, group 2 is absent in invertebrates, is characterized by the presence of a C-terminal SPRY domain and presents unique sets of genes in each mammal examined. The generation of independent sets of group 2 genes is also evident in the other vertebrate species. Comparing the murine and human TRIM sets, we found that group 1 and 2 genes evolve at different speeds and are subject to different selective pressures.

**Conclusion:**

We found that the TRIM family is composed of two groups of genes with distinct evolutionary properties. Group 2 is younger, highly dynamic, and might act as a *reservoir *to develop novel TRIM functions. Since some group 2 genes are implicated in innate immune response, their evolutionary features may account for species-specific battles against viral infection.

## Background

The TRIM gene family encodes proteins involved in a broad range of biological processes and characterized by the presence of the tripartite motif (hence the name TRIM), which consists of a RING domain, one or two B-box motifs and a Coiled-coil region (RBCC) [1,2]. The tripartite motif is always present at the N-terminus of the TRIM proteins. The order of the domains that compose the motif is also conserved: a RING finger domain precedes the B-box motif(s), and a Coiled-coil (CC) region invariably follows. Even if one of the domains is missing, the order of the remaining ones is maintained. Different C-terminal domains are associated with the tripartite motif in the TRIM family [1-4].

Both RING and B-boxes are cysteine-rich zinc-binding domains. The RING finger domain is present, in combination with other domains, in hundreds of proteins and is defined by a linear series of conserved cysteine and histidine residues that represent zinc coordination sites [5]. The B-boxes are the critical determinants of the TRIM family and can be present as B-box1 and B-box2, which share a similar but distinct pattern of cysteine and histidine residues [1]. When both B-box domains are present, type 1 always precedes type 2; when only one B-box domain is present, it is always type 2 [1]. While the tripartite motif is restricted to this protein family, the C-terminal domains are also found in unrelated proteins. A limited choice of C-terminal domains is found in association with the tripartite motif and determined the recent classification of the TRIM proteins in subfamilies [4]. This conserved multi-domain structure appears to behave as an integrated module, rather than a collection of separate motifs, suggesting a possible common function [1,6].

We previously classified 35 human RBCC-containing proteins as a gene-protein family and named it TRIM. We observed that these proteins have strong self-association ability, mainly mediated by their CC region, which results in the formation of large protein complexes. In most cases the TRIM proteins identify different discrete nuclear and/or cytoplasmic sub-cellular structures [1].

The presence of the RING domain and recent experimental evidence indicate that these proteins can act as E3 ubiquitin ligases, the proteins responsible for mediating the transfer of the ubiquitin moiety to the specific targets [7-10]. Alteration of their activity within ubiquitylation processes might be responsible for the clinical manifestation observed in human diseases caused by mutations in TRIM genes [3,6]. *PML*, *RFP*, *TIF*, and *EFP *are implicated in tumor insurgence and progression [11-14]. Other TRIM genes are involved in Mendelian inherited disorders: *MID1 *and *MUL *are altered in two developmental genetic diseases, Opitz Syndrome and Mulibrey nanism, respectively [15,16]. *TRIM32 *is involved in both a form of muscular dystrophy and a form of Bardet-Biedl Syndrome (BBS11), and *MURF-1 *is implicated in muscular atrophy [17-19]. Ro52 is the target antigen of auto-antibodies in both Sjogren syndrome and Systemic Lupus Erythematosus [20]. Finally, TRIM5α has been identified as the major factor restricting HIV-1 during the early phase of infection in Old World monkey cells [3,21].

The TRIM family represents one of the largest classes of putative single protein RING-finger E3 ubiquitin ligases, strongly suggesting that the tripartite motif was selectively maintained to carry out a specialized basic common function within the ubiquitylation process. We used a genomic approach to complete the identification of all human TRIM genes and to study their evolutionary relationships in vertebrate and invertebrate organisms. We observed a general paradigm for the evolution of this family and propose a possible relationship between the evolution of TRIM genes and that of their function.

## Results

### Defining the complete set of TRIM genes in humans and other mammals

To search for all TRIM genes in humans, mouse, rat, cow, and dog, we screened their genomic sequences, using all known mammalian TRIM sequences as queries, with the BLAST and BLAT algorithms at the NCBI and UCSC genome browsers. We also performed a Pattern-Hit Initiated-Blast (PHI-Blast) search against both redundant and non-redundant databases using the sequence patterns that we previously defined for the two B-box domains as query [1]. In addition, we used representative B-box1 and B-box2 sequences to perform TBLASTN genome screening aimed at identifying all the potential loci encoding for B-box-containing proteins. Each retrieved genomic sequence was compared to available EST/cDNA sequences to infer gene architecture. For those genes that lacked a transcript counterpart, we performed a careful manual examination of the genomic sequences by aligning them to the most closely related TRIM of the same or other species to define exon boundaries.

By combining these methods in several iterations, we retrieved the entire set of human TRIM genes. While most of these have been recently reported in the context of other studies, we also report some novel TRIM genes [3,4,22] (http://TRIMbase.tigem.it). Some of the genes we found are present as perfect or almost perfect multiple duplications in the pericentromeric region of chromosome 11 and it is difficult in these cases to establish whether they represent expressed genes (see also below). We also annotated the TRIM complement in mouse, rat, cow, and dog. The inventory of these sets and their comparisons are available at (http://TRIMbase.tigem.it).

### Domain composition of human TRIM proteins

The majority of the human proteins reported in http://TRIMbase.tigem.it fulfill the TRIM rule of domain order and composition (RING, B-box(es), CC, C-terminal domain(s)). During our searches, we also found genes encoding 'incomplete' TRIM proteins, i.e. lacking one of the domains present within the tripartite motif (RING, B-box, or CC). Differently from the RING and CC domains, our analysis clearly indicated that B-box domains are virtually always present within the tripartite motif in metazoans. However, there are a few exceptions in which the B-box(es) domain is associated with only one of the domains belonging to the tripartite motif: in humans 6 proteins that possess B-box(es) lack the RING domain (B-box and CC) and 2 have a very short sequence after the B-box and almost entirely lack the CC region (RING and B-box).

In the evolutionary analyses reported in this study we included the 68 genes listed in Table 1: the 'orthodox' TRIM genes and the 8 'incomplete' TRIM genes or TRIM-like genes (possessing the B-box domain associated with either the CC or the RING domains). The 'incomplete' TRIM-like genes are included in Table 1 and mentioned within the text with their non-TRIM names to remark their non full adherence to the strict definition of TRIM member; within the databases they are also annotated with a TRIM name, which is reported (Table 1). Moreover, of the chromosome 11 pericentromeric clusters we included in the analyses only representative members.

**Table 1:** Human TRIM genes included in our study^a^

	DOMAINS^b^	ACC. No.	MAPPING	Alternative names
TRIM1	R-B1-B2-CC-COS-FN3-SPRY	NM_052817	Xq22.3	MID2
TRIM2	R-B2-CC-IGFLMN-NHL(6)	NM_015271	4q31.3	RNF86, KIAA0517, Narf
TRIM3	R-B2-CC-IGFLMN-NHL(6)	NM_006458	11p15.4	BERP, HAC1, RNF22, RNF97
TRIM4	R-B2-CC-PRY-Spry	NM_033091	7q22.1	RNF87
TRIM5	R-B2-CC-PRY-Spry	NM_033034	11p15.4	RNF88
TRIM6	R-B2-CC-PRY-SPRY	NM_001003818	11p15.4	RNF89
TRIM7	R-B2-CC-PRY-SPRY	NM_203293	5q35.3	GNIP, RNF90
TRIM8	R-B1-B2-CC-nd	NM_030912	10q24.32	GERP, RNF27
TRIM9	R-B1-B2-CC-COS-FN3-SPRY	NM_015163	14q22.1	RNF91, SPRING, KIAA0282
TRIM10	R-B2-CC-PRY-SPRY	NM_006778	6p21.33	RNF9, HERF1, RFB30
TRIM11	R-B2-CC-PRY-SPRY	NM_145214	1q42.13	BIA1, RNF92
TRIM13	R-B2-CC-TM	NM_005798	13q14.2	CAR, LEU5, RNF77, RFP2
TRIM15	R-B2-CC-PRY-SPRY	NM_033229	6p21.33	RNF93, ZNFB7
TRIM17	R-B2-CC-PRY-SPRY	NM_016102	1q42.13	RBCC, terf, RNF16
TRIM18	R-B1-B2-CC-COS-FN3-PRY-SPRY	NM_000381	Xp22.22	MID1, FXY, OSX, XPRF, GBBB1, RNF59, ZNFXY,
TRIM19	R-B1-B2-CC-EXOIII	NM_033238	15q24.1	PML, MYL, RNF71
TRIM21	R-B2-CC-PRY-SPRY	NM_003141	11p15.4	RO/SSA, SSA, RO52, RNF81
TRIM22	R-B2-CC-Spry	NM_006074	11p15.4	STAF50, RNF94
TRIM23	R-B1-B2-CC-ARF	NM_001656	5q12.3	ARD1, RNF46
TRIM24	R-B1-B2-CC-PHD-BROMO	NM_015905	7q33-q34	PTC6, TF1A, RNF82, TIF1A, hTIF1, TIF1ALPHA
TRIM25	R-B1-B2-CC-PRY-SPRY	NM_005082	17q23.2	EFP, Z147, RNF147
TRIM26	R-B2-CC-PRY-SPRY	NM_003449	6p21.33	AFP, RNF95, ZNF173
TRIM27	R-B2-CC-PRY-SPRY	NM_006510	6p22.1	RFP, RNF76
TRIM28	R-B1-B2-CC-PHD-BROMO	NM_005762	19q13.43	KAP1, TF1B, RNF96, TIF1B
TRIM31	R-B2-CC-nd	NM_007028	6p22.1	RING, RNF, HCG1, HCGI, C6orf13
TRIM32	R-B2-CC-NHL(5)	NM_012210	9q33.1	HT2A, TATIP, LGMD2H
TRIM33	R-B1-B2-CC-PHD-BROMO	NM_015906	1p13.2	TIF1g, PTC7, RFG7, TIF1G, FLJ11429, KIAA1113
TRIM34	R-B2-CC-SPRY	NM_021616	11p15.4	IFP1, RNF21
TRIM35	R-B2-CC-PRY-Spry	NM_015066	8p21.2	HLS5, MAIR, KIAA1098, MGC17233
TRIM36	R-B1-B2-CC-COS-FN3-Spry	NM_018700	5q22.3	RNF98, RBCC728, haprin
TRIM37	R-B2-CC-MATH	NM_015294	17q23.2	MUL, TEF3, KIAA0898
TRIM38	R-B2-CC-PRY-SPRY	NM_006355	6p22.2	RNF15
TRIM39	R-B2-CC-PRY-SPRY	NM_021253	6p21.33	RNF23
TRIM40	R-B2-CC	NM_138700	6p21.33	RNF35
TRIM41	R-B2-CC-PRY-Spry	NM_033549	5q35.3	MGC1127, MGC31991
TRIM42	R-B1-B2-CC-COS-FN3	NM_152616	3q23	FLJ40097
TRIM43	R-B2-CC-Spry	NM_138800	2q11.1	
TRIM45	R-B1-B2-CC-IGFLMN	NM_025188	1p13.1	RNF99, FLJ13181
TRIM46	R-B1-B2-CC-COS-FN3-Spry	NM_025058	1q22	FLJ23229, TRIFIC
TRIM47	R-B1-B2-CC-PRY-Spry	NM_033452	17q25.1	GOA, RNF100
TRIM49	R-B2-CC-Spry	NM_020358	11q14.3	RNF18
TRIM50	R-B2-CC-PRY-Spry	NM_178125	7q11.23	
TRIM54	R-B2-CC-COS	NM_032546	2p23.3	RNF30, MURF1
TRIM55	R-B2-CC-COS	NM_184085	8q13.1	RNF29
TRIM56	R-B1-B2-CC-nd	NM_030961	7q22.1	RNF109, DKFZP667O116
TRIM58	R-B2-CC-PRY-SPRY	NM_015431	1q44	BIA2, FLJ38869, DKFZp434c091
TRIM59	R-B2-CC-TM	NM_173084	3q25.33	RNF104, TSBF1
TRIM60	R-B2-CC-PRY-Spry	NM_152620	4q32.3	RNF129, FLJ35882
TRIM61	R-B2-CC-PRY-Spry	NM_001012414	4q32.3	
TRIM62	R-B2-CC-PRY-Spry	NM_018207	1p35.1	FLJ16558
TRIM63	R-B2-CC-COS	NM_032588	1p36.11	RNF28, MURF2, IRIS
TRIM64	R-B2-CC-Spry	XM_061890	11q14.3	
TRIM65	R-B2-CC-Spry	NM_173547	17q25.1	
TRIM67	R-B1-B2-CC-COS-FN3-Spry	NM_001004342	1q42.2	TNL
TRIM68	R-B2-CC-PRY-SPRY	NM_018073	11p15.4	SS-56, DKFZp686D0513, RNF137, FLJ10369
TRIM71	R-B1-B2-CC-IGFLMN-NHL(6)	DQ232881	3p23	hLIN41
TRIM72	R-B2-CC-PRY-Spry	NM_001008274	16p11.2	
TRIM73	R-B2-CC	XM_353628	7q11.23	TRIM50B
TRIM74	R-B2-CC	NM_198853	7q11.23	TRIM50C
TRIM75	R-B2-CC-PRY-SPRY	XM_939332	4q32.3	
KIAA0129	B2-CC-PRY-SPRY	NM_033220	9q22.33	TRIM14, Pub
EBBP	B1-B2-CC-PRY-SPRY	NM_006470	17p12	TRIM16
PYRIN	PAAD-B2-CC-PRY-SPRY	NM_000243	16p13.3	TRIM20, MARENOSTRIN, FMF, MEFV
ATDC	B1-B2-CC-nd	NM_012101	11q23.3	TRIM29
DIPB	B1-B2-CC	NM_017583	11p13	TRIM44
KIAA0298	B1-B2-CC-PHD-BROMO	XM_084529	11p15.4	TRIM66
RNF101	R-B2	NM_024114	11q11	TRIM48, MGC4827
RNF102	R-B2	NM_032765	5q35.3	TRIM52, MGC16175

^a ^The 68 genes listed include 60 TRIM genes and the 8 genes with an incomplete tripartite motif (see text).

^b ^R, RING finger; B1, B-box1 domain; B2, B-box2 domain; CC, Coiled-coil; FNIII, Fibronectin type III repeat; PRY, PRY domain; SPRY domain; Spry, SPRY domain detected only by PFAM; NHL, NHL repeats; IGFLMN, Filamin type immunoglobulin domain; MATH, MATH domain; PHD, Plant Homeodomain; BROMO, BROMO domain; ARF, ARF domain; PAAD, PAAD domain; COS, COS-box; ExoIII, Exonuclease III motif; nd, no known domain detected.

With the identification of the entire complement of the human TRIM and TRIM-like family, we confirmed and extended the domain composition features of these proteins. Within the TRIM modular structure we found that the spacing between adjacent domains is conserved. In fact, the distance between the RING domain and the first B-box, either type 1 or type 2, ranges from 35 to 55 residues; the distance between the two B-box domains ranges from 13 to 20 amino acids; and the spacing, partly occupied by the CC region, between the B-box2 and the C-terminal domain is usually 170–220 residue-long. The maintenance of the domain scaffold, order and spacing clearly indicates that the TRIM structure is a functional module.

#### The Tripartite Motif

We aligned the sequences of the RING finger domain of all human TRIM proteins to define a general TRIM-specific RING pattern (Additional file 1). Besides the cysteine and histidine residues, which coordinate the two zinc atoms, we found clear preferences for specific residues in positions that are probably required to maintain the cross-brace structure of the RING domain [23]. The loop delimited by the second and third Cys residues has a tighter length range within the TRIM family (on average 11 residues) than within other RING-containing proteins [5]. The second loop, bounded by Cys6 and Cys7, is frequently longer than the 48 residues of the general RING consensus [5]. The RING domain has been associated with the ubiquitylation process and is mainly responsible for the interaction with the ubiquitin conjugating enzymes (E2) in the ubiquitylation cascade process [7]. The different length and composition of the intervening sequences of the RING loops may underlie the binding specificity towards the different E2 enzymes.

The comparison of the B-box domains from all TRIM and TRIM-like sequences confirmed that the pattern of Cys and His is similar, although clearly distinct, in the two types of B-boxes (Additional file 1) [1,2]. The B-box1 has a short and tight consensus in which, besides the Cys and His that coordinate two atoms of zinc [24], only two positions show a clear preference for a limited choice of residues. B-box2 sequences are longer than type 1 and their consensus is looser. The cysteine and histidine residues at all 8 possible coordination positions are highly conserved consistent with the recently reported B-box2 structure definition that revealed the coordination of 2 zinc atoms [25] in contrast with previous data [26]. Moreover, additional non-polar or hydrophobic residues are also maintained in defined positions. Twenty-two out of 60 TRIM and 8 TRIM-like proteins possess both B-boxes, with B-box1 always preceding B-box2, whilst the remaining proteins have a single type 2 B-box domain (Table 1).

We observed that the third component of the tripartite motif, the Coiled-coil (CC) region, follows the B-box2 in all bona fide human TRIM proteins as well as in six of the eight TRIM-like proteins. Only RNF101/TRIM48* and RNF102/TRIM52* do not possess this Coiled-coil region as they are truncated immediately after the B-box2. In all other cases, the CC region is always confined within 120 amino acids from the end of the B-box2 domain and in approximately 50% of the human TRIM proteins is bipartite (Additional file 2).

#### TRIM C-terminal domains

The C-terminal domains found in the TRIM and TRIM-like family members are not an exclusive property of this family but are also present in otherwise unrelated proteins [1,4]. The definition of the full complement of human TRIM and TRIM-like proteins allowed us to update the occurrence of C-terminal domains displayed by these proteins (Table 1).

The majority of the human TRIM and TRIM-like proteins (40 members) possess either the SPRY domain or the association of PRY and SPRY domain, also known as B30.2 or RFP-like domain. Table 1 reports the presence of the PRY and SPRY domains that we found using the domain detection tools described in Methods and detailed in the legend but, due to the complicated and still debated relationship between PRY-SPRY and B30.2 domains, we will herein simply refer to them as SPRY [27-29]. The SPRY domain in turn can be associated with Fibronectin type III repeat (FN3) [30] and COS microtubule binding domain in different combinations [4]. Five TRIM proteins display NHL and IGFLMN domains, either in association or alone [31,32]. TRIM56 C-terminal region shares sequence similarity with this domain although no clear NHL repeats are detected. Three TRIM and one TRIM-like proteins contain a PHD associated with a BROMO domain, a combination that was demonstrated to cooperate in nucleosome binding [33]. Other domains are present in only one member of the TRIM family: the MATH domain in TRIM37; the ARF domain in TRIM23; and the EXOIII domain in TRIM19/PML [16,34,35]. Fifteen TRIM and TRIM-like proteins do not possess a defined C-terminal domain. In these cases, either their coding region is limited to the tripartite motif or the C-terminal portion is not similar to any other known domains (Table 1).

This comprehensive review of TRIM and TRIM-like associated C-terminal domains confirmed that a discrete number of motifs have been selected downstream of the tripartite motif in humans.

### The TRIM modular structure is metazoan-specific

**Figure 1:**
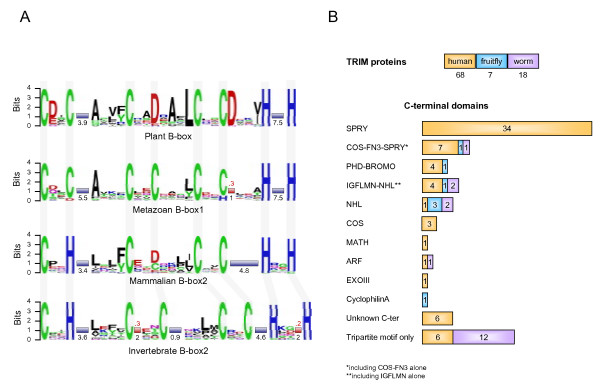
TRIM domains in evolution. **A**) Logo representation of the sequences of Plant B-box (60 B-box sequences; representative species: *A. thaliana*, *O. sativa*, *P. sativum*, *B. nigra*); Metazoan B-box1 (all the B-box1 sequences in representative species: *H. sapiens*, *D. melanogaster*, and *C. elegans*); Mammalian B-box2 (all B-box2 sequences in representative species: *H. sapiens*) and Invertebrate B-box2 (all B-box2 sequences in representative species: *D. melanogaster*, and *C. elegans*). The overall height of each position is proportional to its information content and, within a given position, the conservation of each residue is represented as the relative height of amino acid symbols. Shaded columns indicate the residues involved in the coordination of zinc atoms. Blue bars represent amino acid segments of variable length; the mean value for each segment is reported. Red bars represent segments of fixed amino acid length that are present only in a proportion (indicated in red above the bar) of proteins. **B) **TRIM complements of humans, fruitfly (*D. melanogaster*) and worm (*C. elegans*). The total number of TRIM and TRIM-like genes in each species is indicated (top). The presence of the TRIM associated C-terminal domains is indicated with the same color code (bottom). The length of each bar in the bottom part is proportional to the number (also indicated) of the relative TRIM C-terminal domains found in each species.

To trace back in evolution the origin of the TRIM family, we used human B-box1 and B-box2 sequences as queries to investigate the occurrence of these domains in the genomes of prokaryotic and eukaryotic representative species. We did not find any sequences similar to the B-box domains in prokaryotes. B-box sequences are present in plants with a consensus that is more similar to B-box1 than B-box2 (Fig. 1A). We examined 50 B-box containing proteins from 4 plant species (*A. thaliana*, *O. sativa*, *P. sativum*, *B. nigra*): the B-box is found alone or associated with a second B-box, with the CCT (CONSTANS, CO-like, and TOC1) domain [36], or with both. Differently from mammals, proximal and distal plant B-boxes (we analyzed a total of 60 B-box sequences) are very similar to each other and, consistently, do not separate in distinct branches in phylogenetic analysis (data not shown). No association with RING or Coiled-coil domains was detected in all the plant proteins analyzed.

Besides plants and metazoans, we found B-box domains in some unicellular eukaryotes (unpublished observation). These protist species possess B-box domains that resemble either the plant or metazoan consensi, but the difficulty in attributing these lineages to specific clades compounds the tracing of the evolution of their B-box domain. In addition, since many of these protists are parasites of metazoans, we cannot rule out the possibility that horizontal gene transfer might have occurred [37].

Among the metazoans we also searched the genomes of two invertebrate species, *Drosophila melanogaster *and *Caenorhabditis elegans*, for the presence of B-box domains. Distinct proximal (B-box1) and distal (B-box2) domains are found in these species, sharing with mammals the same B-box1 pattern and a similar B-box2 consensus (Fig. 1A). The B-box domains in these species associate with a RING domain and a Coiled-coil region in a tripartite motif as in mammals. The tripartite motif is therefore exclusive to metazoans, despite the fact that its constitutive elements are not.

However, these invertebrate organisms have TRIM complements that differ significantly from mammals: the fruitfly has 7 TRIM genes and the worm 18, 12 of which code only for a Tripartite motif (Fig. 1B). Fruitfly and worm TRIM proteins share many of the C-terminal domains found in humans, however, their proportion varies among these species, highlighting lineage-specific expansions, e.g. SPRY in humans (Fig. 1B).

### The human TRIM family can be subdivided in two distinct groups: group 1 and group 2

**Figure 2:**
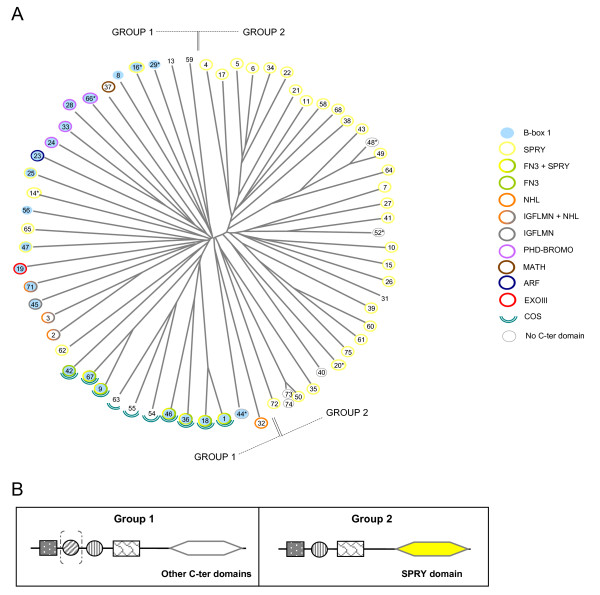
Relatedness of the human TRIM and TRIM-like proteins. **A**) Unrooted phylogenetic tree generated upon alignment of the B-box2 and Coiled-coil region of human TRIM and TRIM-like proteins. The numbers indicate the TRIM family members; numbers with an asterisk indicate the 'incomplete' TRIM proteins named with their alternative TRIM name (see Table 1); light blue circles indicate the presence of the B-box1 domain; the colored open circles represent the different C-terminal domains as indicated in the figure. Partition in groups 1 and group 2 is indicated. **B**) Representative protein structures of the two groups obtained in A); dashed parentheses indicate that B-box 1 may not be present.

Given the numerical and structural complexity of TRIM genes in humans, we sought to characterize the relatedness among members of the family. The presence of different combinations of domains characterized by spaced cysteine and histidine residues rendered a global and reliable alignment of all TRIM and TRIM-like proteins along their entire length difficult. We therefore performed an initial alignment using the B-box2 and Coiled-coil portion. The unrooted phylogenetic tree generated from this alignment supports a recent expansion of the genes that contain the SPRY domain and suggests a preliminary separation of the human TRIM proteins in two main groups based on domain composition and branch topology (Fig. 2). Group 1, composed of 34 proteins (29 TRIM and 5 TRIM-like proteins), includes a high proportion of members with a RING-B1-B2-CC module in combination with all the C-terminal domains found in TRIM proteins. Group 2 is composed of the remaining 34 proteins (31 TRIM and 3 TRIM-like proteins), which possess only the B-box2 domain and are mostly organized as RING-B2-CC-SPRY; the 5 proteins of this group that lack the SPRY domain consist of the tripartite motif alone (Fig. 2).

The alignment of either single or combination of domains that compose the tripartite motif produces similar tree topologies (Additional file 3). This suggests that co-evolution of the domains present within the tripartite motif has occurred, i. e. this module mainly evolved as a single block, with no evidence of large rearrangements leading to domain acquisition or swapping among different TRIM family members; the only exceptions are the incomplete TRIM proteins that have lost one of the tripartite motif domain.

We investigated whether the two groups show differences at the level of their genomic organization. Considering solely the coding region, group 2 genes span on average 10.3 kbp split in 5.7 coding exons compared to the 45.4 kbp and 8.3 exons of the genes that belong to group 1, differences that are statistically significant (*P *< 0.01 for gene length and exon number). Besides the average values, the homogeneity of group 2 with respect to these two parameters is striking. In fact, group 2 gene lengths range from 1.4 to 27 kbp, with only 2 genes larger than 20 kbp, whereas group 1 genes are distributed within a larger range, 1.2 to 143 kbp. Homogeneity of group 2 is also observed for the distribution of the number of coding exons: about two thirds of group 2 TRIM genes are composed of 6 or 7 exons and only in one case they span over 10 exons; again the distribution for group 1 is broader, ranging from 1 to 20 exons.

Taken together, group 2 genes are smaller and less complex than group 1. Interestingly, several group 2 genes are clustered in small chromosomal regions, especially within the Major Histocompatibilty Complex region in 6p21.33 (Table 1 and Additional file 4) [38,39]. The high homogeneity of group 2 genes and their organization in clusters suggest a more recent origin of this group.

### Group 2 TRIM genes evolve more rapidly than group 1

To investigate whether group 1 and 2 have different evolutionary dynamics, we compared the members of the human TRIM and TRIM-like set to their mouse counterparts (see http://TRIMbase.tigem.it and below for the definition of orthologous pairs). Ten out of 60 human TRIM and 8 TRIM-like genes (*TRIM4*, *5*, *22*, *43*, 48*, *49*, 52*, *64*, *73*, *74*) do not have a murine ortholog, whereas TRIM31, 15, 20, and 61 are divergent at the level of entire domains compared to the mouse. Interestingly, these 14 non-conserved or divergent genes fall within group 2 and represent an important proportion of this group (41%). Conversely, all human group 1 genes have a mouse ortholog.

**Figure 3:**
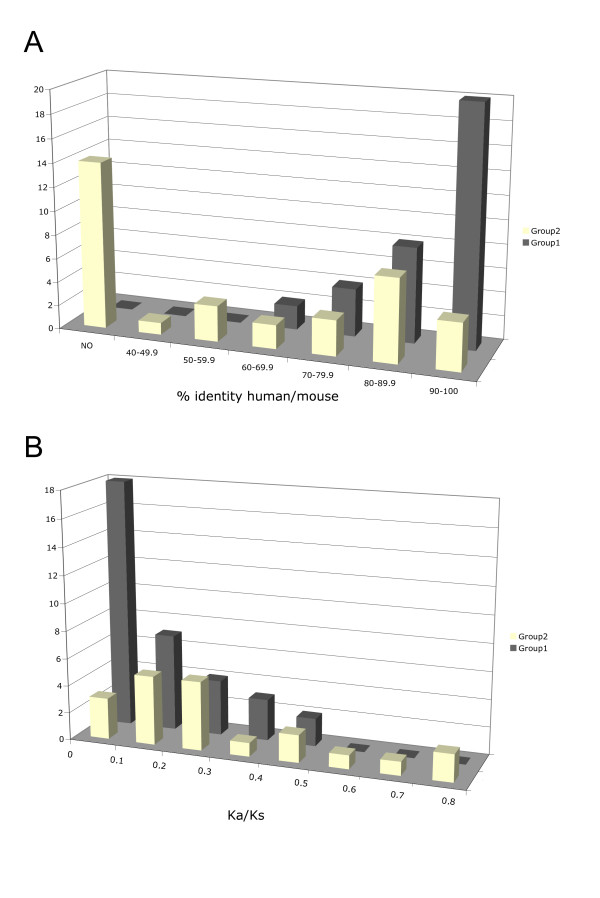
Group 1 and group 2 TRIM gene conservation in human and mouse. **A**) Distribution of the percentage of amino acid identity between human TRIM and TRIM-like proteins and their murine counterparts. Group 2 (yellow) and group 1 (grey). The bars represent the number of human TRIM genes (Y axis) for each percentage of identity interval (X axis); NO indicates absence of a murine counterpart. **B**) Distribution of the Ka/Ks ratios observed in human-mouse orthologous TRIM pairs considering the two groups separately. The bars represent the number of TRIM pairs (Y axis) for each Ka/Ks value interval (X axis).

The degree of conservation of the remaining 54 human/mouse pairs is highly variable, ranging from 49% to more than 99% amino acid identity (peptide comparisons are available at http://TRIMbase.tigem.it). Group 2 pairs present on average 78% amino acid identity versus 89.2% of group 1 (*P *< 0.01). Furthermore, the majority of group 1 proteins show 90–100% amino acid identity against 50–90% of most group 2 proteins (Fig. 3A).

Fast-evolving genes tend to have a higher ratio of nonsynonymous substitutions per nonsynonymous site (Ka) to synonymous substitutions per synonymous site (Ks) than the slow-evolving ones. The average Ka/Ks ratio between human and rodent coding sequences is 0.18 [40]. We analyzed the Ka/Ks ratio in human-mouse TRIM and TRIM-like orthologous pairs and found that group 1 genes present on average a ratio of 0.13 versus 0.29 of group 2 (*P *< 0.01). The Ka/Ks distribution is also significantly different (*P *= 0.01091) between the two groups: the majority of group 1 genes display values below 0.1 while most of group 2 show values above 0.1 (Fig. 3B). Furthermore, only members of group 2 (TRIM38, 40, 60, 75) exceed a Ka/Ks ratio value of 0.5 (Fig. 3B).

Taken together, our analyses indicate that group 2 genes evolve more rapidly compared to group 1. This suggests that the two groups may be subject to different evolutionary constraints, likely underlying species-specific adaptations.

### Group 2 is absent in invertebrates and includes species-specific genes in mammals

**Figure 4:**
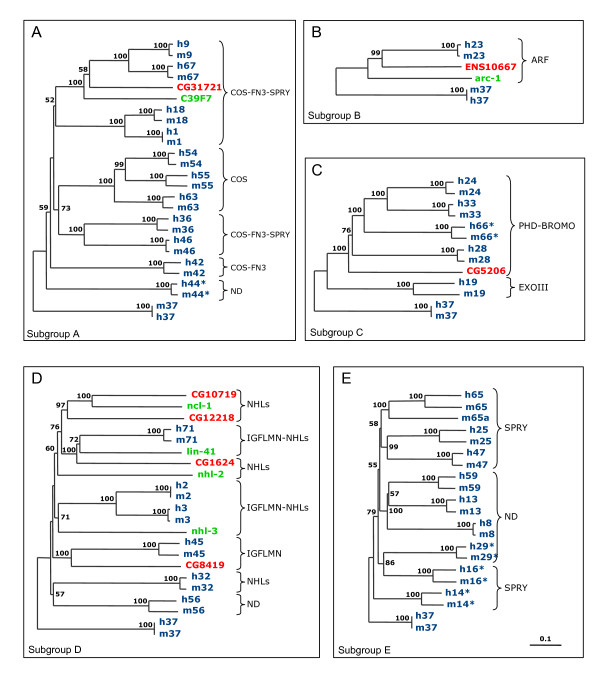
Phylogenetic analysis of human (h, blue), mouse (m, blue), fruitfly (CGs, red), and worm (green) group 1 TRIM and TRIM-like proteins. Human and mouse TRIM proteins are indicated with their TRIM numbers; 'incomplete' TRIM proteins are indicated with their alternative TRIM number with an asterisk (see Table 1); fruitfly and worm sequences are indicated with GenBank accession numbers. Bootstrap support values above 50% based on 1000 replicates are shown. The main domain distal to the tripartite motif is indicated on the right; ND indicates no known domain detected. Panels A-E show the evolutionary relationships among the members of TRIM subgroups that belong to group 1; the worm TRIM genes composed of the R-B2-CC motif only (Figure 2B) represent a separate group related to group 1 and are not shown in the figure. TRIM37 (C-terminal domain: MATH) did not segregated within any subgroups in preliminary analyses and was therefore used as an outgroup in all phylogenetic analyses. The trees were drawn to the scale of amino acid sequence divergence indicated at the bottom right corner. A) Subgroup A includes FN3 and FN3-related TRIM sequences. Fruitfly CG31721 and its worm ortholog C39F7 are the only invertebrate proteins present in the FN3 subgroup and segregate with mammalian TRIM9 and 67. B) Subgroup B includes ARF-related TRIM sequences. Genes encoding a protein homologous to TRIM23 are found in worm and in the honeybee *Apis mellifera *(ENS10667 = ENSAPMT00000010667) but not in *D. melanogaster*, suggesting that the ARF domain has been acquired by a tripartite-gene precursor before vertebrate-invertebrate lineage separation, and has occasionally been lost in some species. C) Subgroup C includes PHD-BROMO and PHD-BROMO-related TRIM sequences. Fruitfly *CG5206 *behaves as an outgroup for all human and mouse PHD-BROMO proteins, suggesting that it may be regarded as an ortholog of their protein ancestor. D) Subgroup D includes IGFLMN-related TRIM sequences. This subgroup is the only example of TRIM expansion in invertebrates, because it includes worm and fly genes that do not have any direct correspondent in mammals. E) Subgroup E includes TRIM proteins with B1, B2, and SPRY in various combinations (see Table 1). No invertebrate sequences are found within this subgroup.

**Figure 5:**
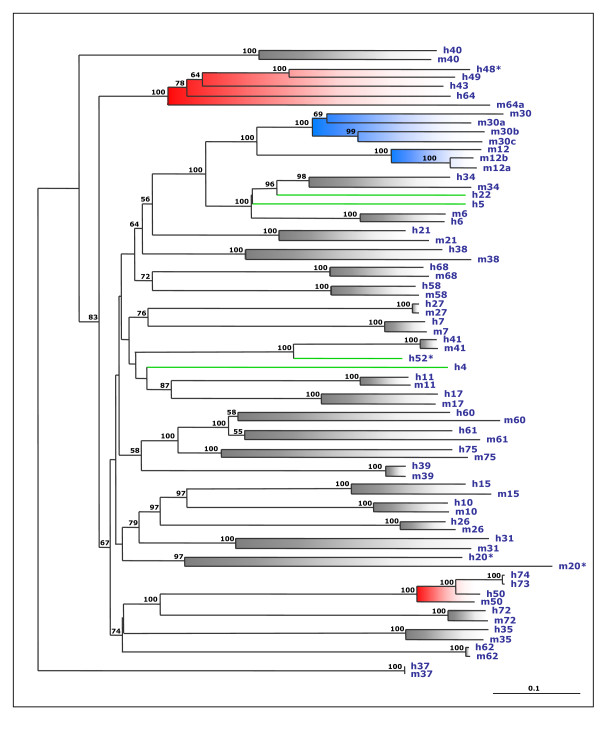
Phylogenetic analysis of human (h) and mouse (m) TRIM and TRIM-like proteins from Group 2. TRIM proteins are indicated with their TRIM number ('incomplete' TRIM proteins are indicated with their alternative TRIM number with an asterisk, see Table 1). No invertebrate TRIM proteins are represented in this group. Bootstrap support values above 50% based on 1000 replicates are shown. Group 1 TRIM37 sequences are used as outgroup. The scale of amino acid sequence divergence is indicated at the bottom right corner. Twenty-four pairs of orthologs can be identified (gray-shadowed branches). The remaining proteins can be subdivided in three different types, based on the phylogenetic relationship with their neighbors: (i) Proteins that are present only in one species and apparently started to diverge from their paralogs before human-mouse split (green clades); (ii) Clades of paralogous proteins that are present only in human and share a single homologous counterpart in mouse (red-shadowed branches); (iii) Clades of paralogous proteins that are present only in mouse and do not have any obvious homologous counterpart in humans (blue-shadowed branches).

Preliminary rounds of sequence alignment and evolutionary analyses allowed us to divide the TRIM and TRIM-like family members into major classes and subsequently generate their phylogenetic trees separately using the full-length protein sequences from man, mouse, fruitfly and worm (see Methods for details). The results show that these proteins are evolutionarily organized in two groups that coincide with group 1 and group 2 (Fig. 4 and 5). The only discrepancy with the previous subdivision is TRIM62, which segregates with group 2 proteins in the evolutionary analysis. Within group 1, TRIM37 did not segregate with any subgroups in preliminary studies and therefore it was used as an outgroup in all phylogenetic analyses.

Group 1 is further divided into subgroups that grossly match with the domains downstream of the tripartite motif (Fig. 4). This analysis confirms that members of group 1 are present in both human and mouse with a strict 1:1 orthologous correspondence and are represented in invertebrates (Fig. 4). By means of this phylogenetic analysis we could appreciate better the mammals-invertebrates TRIM relationship and, since the worm and the fruitfly possess many of the C-terminal domains present in mammals, we could follow the late evolution of the TRIM modular structure as discussed below. Interestingly, no invertebrate sequences are found to be homologous to members of group 1 subgroup E, which includes TRIM and TRIM-like proteins with B1, B2, and SPRY in various combinations (Fig. 4E and Table 1). Group 2 proteins are not represented in invertebrate species as well, and have either a complete or a truncated RING-B2-CC-SPRY domain composition (Fig. 5 and Table 1). The major structural difference between subgroup E and group 2 is the presence of the B-box1, which indicates that group 2 proteins could have derived from a subgroup E member upon loss of B-box1.

Interestingly, the evolutionary analysis of group 2 proteins showed, in addition to 24 pairs of orthologs, the presence of species-specific TRIM and TRIM-like proteins, not only in humans (see above) but also in mouse (Fig. 5), indicating high dynamicity in the evolution of this group compared to group 1.

The analysis of the TRIM and TRIM-like complements of rat, cow, and dog showed that these species have the same set of group 1 genes as humans and mouse. Conversely, the sets of group 2 genes are different and specific to each species, including closely related ones such as mouse and rat, with only 17 genes (50–70% of group 2 genes) shared among all species (http://TRIMbase.tigem.it). These differences are due to events of gene duplication, deletion, or degeneration that likely occurred during the evolution of each single lineage. Remarkably, in three cases the same gene has undergone degeneration or deletion independently in different lineages: human *TRIM38 *orthologs, found in mouse and cow, are pseudogenes in rat and dog; human *TRIM60 *has orthologous loci in all the examined mammals but has become pseudogenes in rat, cow, and dog; human *TRIM61 *orthologs are found to be pseudogenes or absent in all organisms but mouse. Furthermore, two TRIM genes are present in the five species in three different states: *TRIM15 *is intact in human and cow, presents premature stop codons in mouse and rat, and is a pseudogene in dog; similarly, *TRIM31 *is intact in mouse and rat, has distal frameshifts in humans and cow, and is absent in dog. Both premature stop codons and frameshifts result in truncated proteins that have lost the SPRY domain. These losses may represent a degenerative step towards the complete inactivation of these genes.

Of note, massive gene duplications and rearrangements had occurred at the level of several genes of group 2 that cluster in the same chromosomal location (Table 1). A thorough characterization of the TRIM-rich 6p21.33 locus is reported in human and chicken [39,41,42]. Indeed, in addition to the human genes here presented, extra-copies of group 2 genes *TRIM43*, 48*, *49*, and *64 *are clustered at 2q11.1, 11q11.1, and 11q14.3 for a total of 11 predicted genes and 14 pseudogenes. These clustered loci have paralogs, but not orthologs, in some of the other examined mammalian species (http://TRIMbase.tigem.it).

A further example of recent gene evolution is the genomic cluster at 11p15.4 containing *TRIM5*, a gene involved in HIV-1 viral restriction in some primates [21]. This cluster includes *TRIM5 *and *22*, which are currently considered primate-specific, in addition to *TRIM6 *and *34*, which are not [43,44]. By means of comparative and evolutionary analyses (Additional file 5) we found that the entire cluster, including *TRIM5 *and *22*, was present in the last common ancestor of humans, cow, and dog, supporting a common origin for *TRIM5 *and its cow functional ortholog *LOC516599 *rather than evolutionary convergence as previously proposed [43].

### TRIM genes in other vertebrate species

Given the situation in mammals, we asked whether the TRIM complements evolved likewise in other vertebrate species. We searched the databases for TRIM and TRIM-like sequences in representative aves and fish species, chick (*Gallus gallus*) and a pufferfish (*Tetraodon nigroviridis*), respectively. In addition, we included in our analysis the urochordata *Ciona intestinalis*, a representative of the early chordate lineage from which the vertebrates originated. The searches were performed combining different iterations of PHI-BLAST (using the B-box2 pattern) and TBLASTN and BLASTP against nr protein and nucleotide databases at NCBI, starting from both human TRIM proteins and TRIM sequences found in the above species. We found 10 TRIM sequences in ciona, 37 in chick and 58 in pufferfish; all the sequences we retrieved were present in the databases as assessed or predicted genes and only some of them (especially in chicken) were already annotated as TRIM genes (the sequences retrieved for these species are reported at http://TRIMbase.tigem.it). These novel TRIM complements should not necessarily be regarded as complete since in these species there are still regions not yet sequenced or unequivocally assembled. We compared these additional vertebrate sequences to the human sequences using multiple protein alignments and phylogenetic tree constructions using as a paradigm the subdivision into groups and subgroups of Figure 4 and 5. Detailed analyses of these novel genes, at the level of transcript, protein and genomic locus, are required and will be addressed elsewhere, therefore the phylogenetic analyses we present show relationships and are not intended to represent precise evolutionary distances.

**Figure 6:**
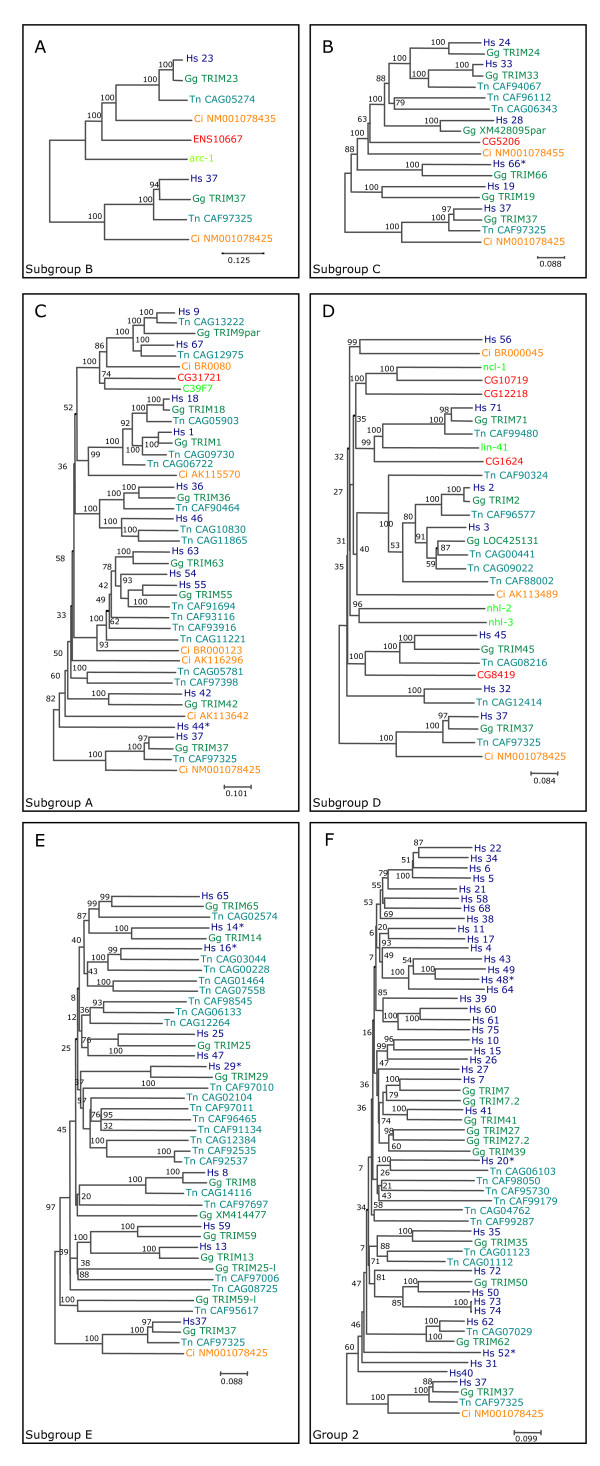
Phylogenetic analysis of TRIM and TRIM-like proteins of representative species of mammals, aves, and fish. Human (Hs, dark blue), chicken (Gg, dark green), tetraodon (Tn, light blue); ciona (Ci, orange), fruitfly (red) and worm (light green) are included. Bootstrap support values based on 1000 replicates are shown. Group 1 TRIM37 sequences are used as outgroups. The scale of amino acid sequence divergence is indicated at the bottom right corner.

In all species analyzed, we found clear orthologs for TRIM23 and TRIM37 (Fig. 6A). With respect to subgroup C, there is one representative of this subgroup in ciona, closely related to the fruitfly member. All the known mammalian members of this subgroup are found in chick with recognizable orthologous relationships (Fig. 6B). This is not completely true for tetraodon in which 3 members belonging to this group have been found: one orthologous to TRIM33 and the others representing fish-specific duplications related to the TRIM33-24 clade. Genes strictly related to KIAA0298/TRIM66* and TRIM19 have not been found in tetraodon (Fig. 6B). In ciona there are 5 representative members of subgroup A, 3 representing the ancestors of different subclades (TRIM9-67; TRIM1-18; TRIM54-55-63) and 2 apparently more ancestral genes. DIBP/TRIM44* appears to be uniquely represented in mammals. All the other members are present in chick and fish although orthology is clear only for TRIM9, 67, 1, 18, and 36. TRIM46, if truly absent, may have been lost in chick while of the MURF group (TRIM54, 55, 63) 2 orthologues are present in chick while 4 members with no direct orthologous relationship are present in fish (Fig. 6C). An analogous situation is observed for subgroup D. In fact, ciona has two representative members; orthologous relationship with human is observed for both chick and fish for four members (TRIM2, 3, 45, 71). TRIM32 might have been lost in chick and TRIM56 in both chick and fish (Fig. 6D). For the above group 1 subgroups, we therefore found that most of the mammalian components are present in chick often with clearly recognizable orthologous relationship. Although the number of members within these subgroups is similar also in tetraodon, the TRIM and TRIM-like genes in this species, consistent with a larger evolutionary distance, have duplicated and diverged more extensively, sometimes obscuring orthologies.

A different situation is observed with the group 1 subgroup E and, as expected, with group 2. As for the other invertebrates, ciona genes are not represented in this subgroup and in group 2. Orthologous relationship among mammals, chick and fish is observed only for two genes of subgroup E (ATDC/TRIM29* and TRIM65). The other genes within the subgroup are more conserved in chicken while in tetraodon many independent duplication events occurred (Fig. 6E). Even more extensive duplication events and independent divergences are observed for group 2 genes. In this case, close homology among the three species is only recognizable for TRIM35 and 62. A couple of other mammalian TRIM genes are conserved in chick (TRIM7 and 41) but the remaining genes in the three species have been subjected to independent duplications and evolution (Fig. 6F).

To confirm in fish the presence of so many TRIM sequences poorly related to the human TRIM genes, we search TRIM and TRIM-like genes also in zebrafish (*Danio rerio*) using the same criteria and methods described for the chick and tetraodon. Differently from tetraodon, zebrafish presents an elevated number of TRIM and TRIM-like genes; we found 240 entries corresponding to independent genes (the list of zebrafish genes is reported at http://TRIMbase.tigem.it). Also in this case, the number of genes encoding for group 1 TRIM (excluding subgroup E) is comparable to the number in mammals, chick and pufferfish (1 subgroup B; 5 subgroup C; 16 subgroup A; 12 subgroup D) although in many cases clear duplication events occurred. However, the great expansion of the TRIM genes in the zebrafish is associated with members belonging to the group 1 subgroup E and group 2 genes (data not shown).

Analyses of TRIM complements in aves and fish corroborate the high conservation of group 1 genes during evolution and highlight the generation of unique sets of group 2 genes in each vertebrate species analyzed. Moreover, the data in non-mammalian vertebrates, especially in tetraodon, confirm that members of the group 1 subgroup E very likely gave rise to group 2 genes.

In conclusion, our study indicates the presence of two distinct TRIM gene groups. Group 1 is evolutionary more ancient than group 2 and is likely to contain basic functions that are essential to both vertebrate and invertebrate species. On the other hand, group 2 is younger and more dynamic, possibly acting as a sort of TRIM genes "reservoir" to develop novel species-specific functions.

## Discussion

Here, we report the identification and genomic characterization of the full complement of the human TRIM family examined from an evolutionary perspective by comparison with several vertebrate and invertebrate species.

We definitively assessed that the B-box domain is only present within the tripartite module in metazoans, with the few exceptions mentioned above and discussed below, and is therefore the defining domain of the TRIM family. We redefined the B-box1 and B-box2 consensi as well as the TRIM specific RING finger pattern using all human sequences. Within these domains we found conservation not only of the residues putatively involved in metal coordination, but also of other amino acids that compose the novel consensi. It will be interesting to model the RING, B-box1 and B-box2 sequences of the TRIM proteins on these structures to study the possible role of the conserved residues in relation to the ubiquitylation cascade [7-9,24,25].

**Figure 7:**
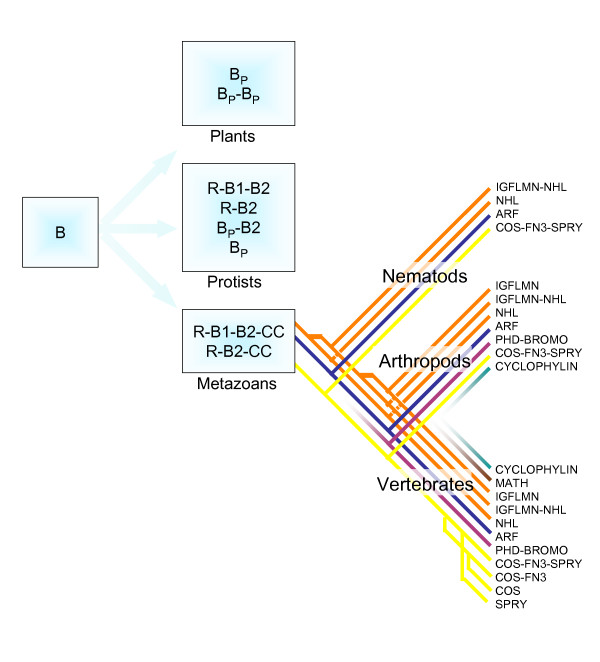
Proposed model for TRIM structure evolution (see text). The C-terminal domains probably derived from a single ancestor domain are indicated with the same color.

Based on our analyses, we propose a general model of TRIM structure evolution (Fig. 7). Our studies suggest that the origin of the B-box domain is quite ancient and probably dates back to a common ancestor of plants and metazoans. The plants maintained either one or two B-boxes without apparent sequence differentiation into proximal and distal. Conversely, metazoans differentiated a pair of B-boxes into a proximal and a distal type. In an early step of metazoan evolution, a RING domain and a Coiled-coil region associated with the B-box(es) to generate a solid tripartite module that has been maintained from invertebrates to mammals. From then onward, the tripartite motif has evolved as a unique block and it is frequently encoded by a single exon. Interestingly, we found different mammalian and invertebrate B-box2 patterns. This may underlie functional coupling with specific interactors in each lineage, which would have forced convergence at specific sites. A similar species-specific evolutionary convergence was recently described for sulfatase enzymes and their common post-translational modification factor [45].

Before invertebrate-vertebrate lineage split, the tripartite module has been associated with a discrete number of C-terminal domains. Addition and loss of C-terminal domains and structure remodeling have then occurred in the various evolutionary lineages (Fig. 7). In at least one case the same domain acquisition has occurred independently in primates and arthropods. In fact, in some species of New World monkeys (*Aoutus *genus), Cyclophilin A (CypA) is fused with the tripartite motif of TRIM5 [46]. TRIM5 confers a potent block to HIV-1 infection in Old World primates, while Cyclophilin A (CypA) enhances infection by direct interaction with the HIV capsid [21,47]. HIV-1 blockage in *Aoutus *cells was explained by the exclusive presence of the *TRIM5-CypA *chimeric gene [46,48,49]. Interestingly, we found that a tripartite motif is associated with a Cyclophilin domain also in fruitfly CG5071 indicating evolutionary convergence. Independent events of gene fusion are considered a hallmark of functional coupling that can be also present in those species in which a similar gene fusion is not observed [50]. Similar processes of fusion between functionally associated domains may have been one of the mechanisms underlying the selection of C-terminal domains during TRIM evolution.

The early association of the B-box modules with the RING finger, a domain linked to the ubiquitylation process, has eventually brought the proteins possessing a tripartite motif to exert a common basic biochemical function, i.e. ubiquitin ligase [6]. The large number of proteins belonging to this family in mammals highlights the success of this module to undertake its task. Since the TRIM family represents one of the largest RING finger classes, it is tempting to speculate that, among the myriads of cellular E3 substrates, a large proportion is demanding the unique tripartite structure for reasons yet to be discovered. Whereas the tripartite motif may provide the catalytic E3 activity and the ability to form the scaffold of the TRIM-defined sub-cellular compartments [1], the C-terminal region may contribute to select the specific substrate and/or direct the tagged substrate towards downstream pathways. The PHD-BROMO domain, for example, determines the association with chromatin, and the TRIM and TRIM-like proteins containing the PHD-BROMO domain are consistently involved in chromatin remodeling [51,52]. Along the same way, MID1/TRIM18 and the related TRIM proteins that possess a COS microtubule-binding domain exert their role on the cytoskeleton [4].

It should be reaffirmed that not all the proteins we included in our study retain an entire tripartite motif. As mentioned in the 'Results section', we decided to include all the genes encoding for proteins with B-box motifs and that in human correspond to the 'complete' TRIM proteins (with RING, B-box and CC) and few 'incomplete' TRIM proteins (or TRIM-like) presenting only two of the three tripartite motif composing domains (with either 'RING and B-box' or 'B-box and CC'). This might pose a formal problem on what should be classified as a TRIM protein. In the classic definition, a protein family is composed of proteins that have a common phylogenetic origin and share a degree of amino acid identity/similarity above an established threshold. In the case of the TRIM family, the initial definition of family was based on the observation that most members of this protein family share the tripartite arrangement at their N-terminal portion [1]. What was not clear was whether the TRIM proteins had a common origin or rather they were the result of domain swapping from evolutionarily unrelated proteins. Our analyses demonstrate that all the proteins with a RING-B-box-CC module actually have a common evolutionary origin. Therefore, what was raised as an 'operative' definition is now demonstrated to be perfectly adherent to the classic definition of a gene/protein family. Our data allow the same conclusion to be drawn for the 'incomplete' TRIM proteins which possess the B-box motif and which we found evolutionarily belonging to the TRIM family. In support of that, some of the 'incomplete' TRIM proteins also present C-terminal domains characteristic of the 'complete' TRIM proteins. This parallel cannot be used for other domains present within the tripartite motif, e.g. the RING domain has been 'used' to build many different protein families in association with several TRIM unrelated domains [22]. On the other hand, we think that a strict definition of TRIM family based only on function is not feasible at present. As discussed above, the presence of the RING domain suggests a role as E3 ubiquitin ligases for the TRIM proteins. Experimentally, this has been proven for some TRIM proteins and we cannot exclude that some of them, although containing the RING domain in the proper tripartite motif, might have a different biochemical role. What is the role of the 6 RING-less proteins we included in our study? They may be involved in ubiquitylation as well by, for example, acting as regulators of orthodox TRIM proteins through hetero-interaction. Given that the recent solution of the B-box1 and B-box2 domains revealed a strong structural similarity with the RING domain [24,25], it is tempting to speculate that these domains may interact with components of the ubiquitylation machinery and attribute these RING-less proteins the role of E3 ubiquitin ligases. Coherently with these observations, we propose to include within the TRIM family all the proteins that are phylogenetically related to established TRIM members and that have a tripartite motif at their N-terminus, including the few examples in which part of this motif has been lost.

The relatively small number of TRIM genes in lower eukaryotes compared to mammals suggests rapid and recent changes of the TRIM family. Our study revealed the presence of two main groups of mammalian TRIM genes that show distinct evolutionary features and that we named group 1 and group 2. Group 1, that is in turn subdivided in several subgroups, is composed of genes that are present in human, mouse, rat, dog, and cow with a one to one relationship. Although orthology with mammals is not always recognizable, this group of genes is highly conserved also in other vertebrates (chick and fish) in number and structure. Our data on the Ka/Ks ratio of human and mouse group 1 genes suggest that they are subject to purifying selection aimed at conserving their function. It is conceivable that group 1 consists of diversified and essential TRIM and TRIM-like functions for which little or no redundancy is present. Consistently, many group 1 genes are involved in basic cellular processes, such as cell cycle progression and transcriptional regulation, and result, when mutated, in developmental disorders, muscular phenotypes, cancer insurgence, etc. [2,6]. Some group 1 TRIM genes have also been found to be involved in viral response, namely *TRIM1 *[53], *TRIM19/PML *[3,54,55] and *TRIM32 *[56]. TRIM19/PML, besides its involvement in acute promyelocytic leukemia, has been shown to interfere with the replicative cycle of many DNA and RNA viruses and evidence indicate that it may represent a broad-spectrum cellular defence factor [3].

The important increase of TRIM number in vertebrates is primarily due to the buildup of the genes that constitute group 2. Group 2 is in fact evolutionarily more recent than group 1, is not represented in invertebrates, and evolves at a faster rate. Interestingly, many TRIM proteins that belong to group 2 have been recently associated with cellular innate immunity towards viral infection. In addition to *TRIM5α*, other members are being investigated as potential retrovirus restriction factors. Among them, *TRIM21*, *22 *and *34 *are regulated by interferons, a family of secreted proteins that exert antiviral and immunomodulatory activities [57]. Moreover, other group 2 genes, *PYRIN*/TRIM20* and *TRIM21 *are involved in immuno-related diseases [20,58,59]. Interestingly, group 2 proteins share the same C-terminal motif, the SPRY domain. In the case of TRIM5α, this domain is responsible for the species-specific HIV-1 restriction and is subject to positive selection in primates, underlying its possible role in directing and specifying capsid recognition [44,60]. Of note, the SPRY domain is also present in SOCS proteins, involved in cytokine signaling and innate immunity, and in the BTN family of lymphoid expressed proteins, possibly involved in immune regulation [28,61,62]. It has been proposed that the sharing of the SPRY domain between the TRIM and BTN family members located within the MHC locus is somewhat linked to their immunological function [41]. The SPRY domain might therefore confer to group 2 proteins the ability to specifically recognize viral capsids and interfere with early steps of viral infection. Differently from anti-viral group 2 proteins, group 1 TRIM19/PML interferes with general mechanisms of viral replication common to various viruses and consistently is not subject to positive selection [35].

Our comparative analysis in five mammalian species shows that subsets of group 2 TRIM and TRIM-like genes are different and specific in each examined lineage. This is more evident in the three non-mammalian vertebrates analyzed, where large numbers of newly identified group 2 genes mainly lie on species-specific evolutionary clades. This observation might underlie dispensability/redundancy of some group 2 genes, which could have provided the basis for novel species-specific roles during evolution. The presence of clusters composed of massively duplicated group 2 genes, in mammals but also in chick, suggests that they may be hot-spots for TRIM gene production and remodeling. In the case of the teleost fish species some of the duplications may be the remnants of the whole genome duplication event early in the teleost lineage. Global duplication is not however enough to explain the large and independent expansion of subgroup E and group 2 genes in the teleosts and a different cause must underlie these expansions. It is interesting to note that similar to the Group 2 TRIM and TRIM-like genes, other families of genes involved in innate immune response and in particular the components that interact with pathogens have been subject to similar large lineage specific expansions in the teleost fish [63]. Moreover, since we observed that group 2 genes tend to have a human/mouse Ka/Ks ratio higher than group 1 genes, it is tempting to speculate that some group 2 genes other than *TRIM5α *may be subject in some species to positive selection at specific sites to counteract species-specific battles against viral infections, as it has been shown for other family of genes involved in innate cellular immunity [64-66].

## Conclusion

We found that the TRIM domain structure is an innovation of metazoans. The growing evidence for a common biochemical function of the TRIM proteins as ubiquitin ligases justifies the maintenance of their basic modular structure throughout evolution. Our studies indicate the presence of two distinct TRIM gene groups. Group 1 is evolutionary more ancient than group 2 and is likely to contain basic functions that are essential to both vertebrate and invertebrate species. On the other hand, group 2 is younger and more dynamic, possibly acting as a sort of TRIM genes "reservoir" to develop novel functions. Since some of the TRIM genes that belong to this group are implicated in innate immune response, we propose that the different selection we observed for this group of genes underlies pressure towards rapid changes necessary to counteract species-specific battles against viral infection.

## Methods

### Gene searching

Known mammalian TRIM and TRIM-like gene/protein sequences were retrieved from the National Center for Biotechnology Information (NCBI) and re-defined by searching against their respective genome assemblies using BLAT at the UCSC genome browser (http://genome.ucsc.edu). All the 'corrected' sequences were then used as queries to search potential novel TRIM genes within the human, mouse, rat, cow, and dog genomes, using BLAT at UCSC and TBLASTN at NCBI [67] genome browsers, respectively. All searches have been performed in several iterations using default parameters. Human nr and EST databases were screened using the PHI-BLAST in several iterations using the patterns previously defined for B-box1 and B-box2 [1]. We also searched the human, mouse, rat, cow, and dog proteomes using the B-box2 as a bait in five iterations of the PHI-BLAST program [67], which provides a highly sensitive analysis, taking advantage of the fact that the B-box2 is a peculiar constituent of TRIM proteins and does not produce a large amount of background noise in this analysis. The retrieved amino acid sequences were subsequently used to search the respective genomes for identifying their encoding loci. Representative B-box2 sequences were also used as queries for TBLASTN search of the five mammalian genomes to identify all the potential loci encoding TRIM and TRIM-like proteins. All the retrieved genomic sequences were aligned to the available cDNA/EST sequences to infer the gene architectures. For genes that lacked a transcript counterpart in public databases, we performed a careful manual examination of the genomic sequences BLAST-comparing them to the putative more closely related ortholog or paralog, looking for splicing donor and acceptor signals to define the exon-intron boundaries. Constructed open reading frames (ORFs) were conceptually translated into amino acid sequences and checked against their closest homologs. The original genome sequencing traces (Traces-WGS), which are available at the NCBI web site, were checked when the constructed coding sequences presented either stop codons or ORF frame-shifts. When a difference between WGS traces and the genomic assembly was evident, the constructed sequence was properly corrected. Comparison of human, mouse, rat, cow, and dog orthologous TRIM and TRIM-like genes showed that >99% of splicing acceptor and donor sites were conserved at the same relative position in the coding sequence in all species, i.e. the gene structure of TRIM genes is identical among different mammals. To retrieve TRIM genes form ciona (*Ciona intestinalis*), chick (*Gallus gallus*), pufferfish (*Tetraodon nigroviridis*) and zebrafish (*Danio rerio*) we used a combination of PHI-BLAST and TBLASTN against nr protein and nucleotide databases at NCBI, respectively. The following genome releases have been used for this work: *Homo sapiens*, May 2004 assembly (NCBI Build 35); *Mus musculus*, February 2006 assembly (NCBI Build 36); *Rattus norvegicus*, June 2003 assembly (Baylor College of Medicine HGSC v.3.1); Bos taurus, March 2005 assembly (Baylor College of Medicine Btau_2.0); *Canis familiaris*, May 2005 assembly (Broad Intitute CanFam2.0). *Drosophila melanogaster *TRIM genes: *CG1624*, *CG5206*, *CG12218*, *CG8419*, *CG5071*, *CG10719*, *CG31721*. *Caenorabditis elegans *TRIM genes: arc1, B0281, ZK1240.1, F43C11.8, ZK1240.2, F43C11.7, ZK1240.9, ZK1240.3, ZK1240.8, ZK1240.6, C28G1.6, K09F6.7, lin41, C39F7, nhl-2, nhl-3, ncl-1, F47G9.

### Protein domain analyses

To identify and analyze the domain composition of the TRIM and TRIM-like protein products we used the major alternative splicing isoforms, if more than one was available, and utilized different domain prediction programs. First, we submitted the TRIM amino acid sequences to the SMART tool [68] where we analyzed the sequence against contemporary the SMART and Pfam [69] domains databases. The denotation of the C-terminal domains found within the TRIM sequences are the following: MATH, SM00061; PHD, SM00249; BROMO, SM00297; IGFLMN, SM00557; EXOIII, SM00479; FN3, SM00060; PRY, SM00589; SPRY, SM00449; ARF, SM00177; NHL, PF01436 (Pfam). The tripartite motif domains were additionally analyzed as described below. Besides the SMART results, the RING and B-boxes domains were also defined in each TRIM and TRIM-like protein by hand using the previously published patterns [1]. In order to obtain a new profiling, the sequences corresponding to each domain were then aligned using the PRATT 2.1 program [70] and the best scoring consensi were selected and integrated by hand. The order of the sequences in the alignment shown reflects their degree of sequence conservation. The region of each TRIM and TRIM-like protein immediately after the last Cys or His of the B-box2 domain was analyzed for Coiled-coil prediction with the Coil 2.2 program [71]. Analysis was performed with MTIDK and MTK matrices and both the weighted option, which takes into account the polarity of the residue within the predicted Coiled-coil heptad repeat, as well as the unweighted option. When differences of around 20–30% in Coiled-coil prediction were observed between the different methods utilized, the prediction was considered bad and not indicated in the list of Additional file 2. Moreover, only percentages of prediction higher than 50% were considered using two residue windows, 21 and 28 amino acids.

Plant B-box containing proteins (from *A. thaliana*, *O. sativa*, *P. sativum*, *B. nigra*) were retrieved from the SMART B-box database [68].

### Evolutionary analyses

To perform phylogenetic analysis, TRIM and TRIM-like protein sequences were aligned using MultAlin [72] in a multi-step process. Only proteins containing the complete module R-B1-B2-CC were aligned in a first step, eliminating from each sequence the portion downstream of the coiled-coil domain and all segments that caused a gap to interrupt the alignment. A first phylogenetic tree was produced starting from this multi-alignment. In a successive step, each of the remaining protein sequences was singularly added to the multi-alignment, edited for exceeding amino acids, and assigned to a TRIM subgroup after inspection of the topology of the resulting phylogenetic tree. Once all TRIM and TRIM-like protein sequences were assigned to a subgroup, phylogenetic analyses were performed independently for each subgroup. TRIM37 was not included in any subgroup and therefore was used as an outgroup in all final analyses. Nucleotide sequences of TRIM5/6/22/34 and related non-human sequences were also aligned using MultAlin [72], but in this case a gap-removal step was not necessary due to the high similarity among all considered sequences. Neighbor-Joining and bootstrap analyses were performed with Phylo_win [73], computing the distances among sequences with all the methods available in the package (protein analysis: observed divergence with and without Poisson correction; PAM distance. DNA analysis: observed divergence; Jukes and Cantor distance; Kimura distance; Tajima and Nei distance; HKY distance; Galtier and Gouy distance; and LogDet distance) [73] (and references therein). Gap-removal was set as pairwise rather than global to minimize information loss. Bootstrap values were computed over 1000 repetitions. All tree topologies resulted to coincide in the different methods for branches with a bootstrap value >50. Evaluation of Ka/Ks values for pairs of human-mouse TRIM- and TRIM-like-coding sequences was performed at the Norwegian bioinformatics platform [74].

Comparison of the two groups quantitative parameters (gene lengths, exon number, amino acid identity and Ka/Ks ratios) were analyzed using the two samples t-test (two-tail test assuming unequal variances) by the Microsoft Excel statistical package. The comparison of the two groups Ka/Ks distribution has been analyzed using a two-sample Kolmogorov-Smirnov test.

## Authors' contributions

MS carried out the evolutionary studies and contributed to the identification of TRIM genes in different species; he contributed to the design of the experiments and to the writing of the manuscript. SC identified the entire set of TRIM genes in human and performed the single domains alignments. BF collected all the data in the database and performed statistical analyses. AB contributed to the interpretation of the data and to the drafting the manuscript. GM conceived, designed and coordinated the study and wrote the manuscript.

## Additional files

The complete sets of human, mouse, rat, dog and cow TRIM and TRIM-like genes and pseudogenes as well as their sequence comparisons are available at http://TRIMbase.tigem.it. At the same site are the TRIM related sequences from ciona, chick, tetraodon, and a list with the accession numbers of the zebrafish TRIM-like genes. See Additional files 1 to 5.

## Supplementary Material

Additional file 1 Includes the alignments of the RING, B-box1 and B-box2 domains of all the human TRIM and TRIM-like proteins, alignments from which the consensi for these domains have been generated. Click here for file

Additional file 2 Reports the values of Coiled-coil predictions for all the human TRIM and TRIM-like proteins. Click here for file

Additional file 3 Shows the unrooted phylogenetic trees generated from the alignments of single domains of the tripartite motif. Click here for file

Additional file 4 Shows a schematic representation of the human TRIM genomic clusters. Click here for file

Additional file 5 Shows comparative and evolutionary analyses of the cluster of *TRIM5*, *6*, *22*, and *34 *in mammals. Click here for file
