# Supplementary material for: Genomic analysis of the TRIM family reveals two groups of genes with distinct evolutionary properties
Source: BMC Evol Biol. 2008 Aug 1;8:225. doi: 10.1186/1471-2148-8-225 (PMC2533329; doi:10.1186/1471-2148-8-225)
Supplement: Additional file 1 — Includes the alignments of the RING, B-box1 and B-box2 domains of all the human TRIM and TRIM-like proteins, alignments from which the consensi for these domains have been generated. [file 1471-2148-8-225-S1.pdf]

A

RING DOMAIN

1 2 3 4 5 6 7 8

$\mathbf{C}xx\mathbf{C}x_{(9-16)}\mathbf{C}x\mathbf{H}xx\mathbf{C}xx\mathbf{C}x_{(7-136)}\ \mathbf{C}xx\mathbf{C}$

$\mathbf{C}x(ILV)\mathbf{C}x_{(2-4)}(FLYI)x_{(2-7)}(PAS)x_{(2-3)}(ILVT)x\mathbf{C}x\mathbf{H}x(FLYVI)\mathbf{C}xx\mathbf{C}(AFILV)x_{(6-135)}\mathbf{C}(PF)x\mathbf{C}$

```
TRIM65: lt CaICl-glFqd-----Pvt-LpCgHnFCgaCIrdwwdrcg-----------------kaCPeC re
TRIM43: lt CvICl-nyFvd-----Pvt-IcCgHsFCrpCLclsweeaqsp---------------anCPaC re
TRIM48*:lt CpICm-nyFid-----Pvt-IdCgHsFCrpCFylnwqdipil---------------tqCFeC ik
TRIM49: li CpLCm-nyFid-----Pvt-IdCgHsFCrpCFylnwqdipfl---------------vqCPeC tk
TRIM64: li CcICv-nyFid-----Pvt-IdCgHsFCrpCLclcseegram---------------prCPsC rk
TRIM11: at CaICl-dyFtd-----Pvm-TdCgHnFCreCIrrcwgqpegp---------------yaCPeC re
TRIM39: as CsVCl-eyLke-----Pvi-IeCgHnFCkaCItrwwedlerd---------------fpCPvC rk
TRIM7:  at CsICl-elFre-----Pvs-VeCgHsFCraCIgrcwerpgagsvgaatrappfp---lpCPqC re
TRIM27: tt CpVCl-qyFae-----Pmm-LdCgHnICcaCLarcwgtaetn---------------vsCPqC re
TRIM17: at CsICl-dyFtd-----Pvm-TtCgHnFCraCIqlswekargkkgrrkrkgs------fpCPeC re
TRIM4:  lt CpICl-dyFqd-----Pvs-IeCgHnFCrgCLhrnwapgggp---------------fpCPeC rh
TRIM52*:av CaICl-dyFkd-----Pvs-IsCgHnFCrgCVtqlwskedeedqneeeed-/112/-ftCPqC rk
TRIM5:  vt CpICl-elLtq-----Pls-LdCgHsFCqaCLtanhkksmldkge------------ssCPvC ri
TRIM6:  vt CpICl-elLte-----Pls-IdCgHsFCqaCItpngresvigqege-----------rsCPvC qt
TRIM34: vt CpICl-elLte-----Pls-LdCgHsFCraCItvsnkeavtsmggk-----------ssCPvC gi
TRIM22: vt CpICl-elLte-----Pls-LdCgHsFCqaCItakikesviisrge-----------ssCPvC qt
TRIM21: vt CpICl-dpFve-----Pvs-IeCgHsFCqeCIsqvgkggg-----------------svCPvC rq
TRIM58: ar CpVCl-dfLqe-----Pvs-VdCgHsFClrCIsefceksdgaqggv-----------yaCPqC rg
TRIM68: va CpICm-tfLre-----Pms-IdCgHsFChsCLsglweipgesqnwg-----------ytCPlC ra
TRIM38: at CsICl-s-Lmtn----Pvs-InCgHsYChlCLtdffknpsqkqlrqet---------fcCPqC ra
TRIM10: vn CpICq-gtLre-----Pvt-IdCgHnFCraCLtryceipgpdlees-----------ptCPlC ke
TRIM15: pa CtLCa-gpLed-----Avt-IpCgHtFCrlCLpalsqmgaqssgki-----------llCPlC qe
TRIM26: vt CsICl-dyLrd-----Pvt-IdCgHvFCrsCttdvrpisgsr--------------pvCPlC kk
TRIM31: vi CpICl-diLqk-----Pvt-IdCgHnFCpqCItqigetscgf---------------fkCPlC kt
TRIM40: gv CpICq-esLke-----Avs-TnCgHlFCrvCLtqhvekasasgv-------------fcCPlC rk
TRIM60: ss CpICl-eyLkd-----Pvt-InCgHnFCrqCLsvswkdlddt---------------fpCPvC rf
TRIM61: as CpICl-dyLkd-----Pvt-IsCgHnFClsCIimswkdlhds---------------fpCPfC hf
TRIM75: ak CsICl-dyLsd-----Pvt-IeCgHnFCrsCIqqswldlqel---------------fpCPvC rh
TRIM50: lq CpICl-evFke-----Plm-LqCgHsYCkgCLvslschldae---------------lrCPvC rq
TRIM73: lq CpICllevFke-----Slm-LqCgHsYCkgCLvslsyhldkt---------------vrCPmC wq
TRIM72: ls CpLCl-qlFda-----Pvt-aeCgHsFCraCLgrvagepaadgt-------------vlCPcC qa
TRIM59: lt CpICy-siFed-----Prv-LpCsHtFCrnCLenilqasgnfyiwrplrip------lkCPnC rs
TRIM13: lt CpICc-slFdd-----Prv-LpCsHnFCkkCLegilegsvrnlswrpap--------fkCPtC rk
TRIM54: li CpICl-emFsk-----PvviLpCqHnLCrkCAndvfqasnplwqsrgsttvssggr-frCPsC rh
TRIM55: li CpICl-emFtk-----PvviLpCqHnLCrkCAsdifqasnpylptrggttmasggr-frCPsC rh
TRIM63: li CpICl-emFtk-----PvviLpCqHnLCrkCAndifqaanpywtsrgssvsmsggr-frCPtC rh
TRIM1:  lt CpICl-klFed-----Pll-LpCaHsLCfsCAhrilvsscssgesiepita------fqCPtC ry
TRIM18: lt CpICl-elFed-----Pll-LpCaHsLCfnCAhrilvshcatnesvesita------fqCPtC rh
TRIM9:  lk CpVCg-sfYre-----Pii-LpCsHnLCqaCArnilvqtpesespqshraa-/72/-itCPqC hr
TRIM67: lk CpVCg-slFre-----Pii-LpCsHnVClpCArtiavqtpdggsaagglgg-/66/-itCPqC hr
TRIM36: li CpaCk-elFth-----Pli-LpCqHsIChkCVkellltlddsfndvgsdns-/36/-fpCPgC eh
TRIM46: ll CpVCq-emYkq-----Plv-LpCtHsVCqaCArevlgqqgyighggdpsse-/51/-fpCPaC qg
TRIM2:  li CsICl-erYkn-----Pkv-LpClHtFCerCLqnyipahslt---------------lsCPvC rq
TRIM3:  lv CsICl-drYqc-----Pkv-LpClHtFCerCLqnyipaqslt---------------lsCPvC rq
TRIM71: qi ClLCkem-/39/------v-LpClHaFCrpCVleahrlpaagggaageplk------lrCPvC dq
TRIM56: la CkICl-eqLra-----Pkt-LPClHtYCqdCLaqladggr-----------------vrCPeC re
TRIM24: dt CaVCh-qnIqsra---Pkl-LpClHsFCqrCLpapqrylmlpapmlgsaet-/22/-irCPvC sq
TRIM33: dt CaVCq-qsLqsrreaePkl-LpClHsFClrCLpeperqlsvpipggsngdiqqvgv-irCPvC rq
TRIM28: eh CgVCr-erLrpere--Prl-LpClHsaCsaCLgpaapaaanssgdggaagdgtv---vdCPvC kq
TRIM45: th CpLCl-glFka-----Prl-LpClHtVCttCLeqlepfsvvdirggdsdts-/19/-ilCPvC da
TRIM19: lr CqqCq-aeakc-----Pkl-LpClHtLCsgCLeasg---------------------mqCPiC qa
TRIM37: fr CfICm-ekLrd-----Arl---CpHCsklCCfsCIrrwlteqr--------------aqCPhC ra
TRIM23: le CgVCe-dvFslqgdkvPrl-LlCGHtVChdCLtrlplhgra----------------irCPfD rq
TRIM35: ll CaVCy-dpFrd-----Avt-LrCgHnFCrgCVsrcwevqvs----------------ptCPvC kd
TRIM42: ln CpmCs-r–Lrlh----Sfm-LpCnHsLCekCLrqlqkhaevtenffi----------liCPvC dr
TRIM62: ll CsICl-siYqd-----Pvs-LgCeHyFCrrCItehwvrqeaqga-------------rdCPeC rr
TRIM8:  li CpICl-hvFve-----Pvq-LpCkHnFCrgCIgeawakdsgl---------------vrCPeC nq
TRIM25: ls CsICl-epFke-----Pvt-TpCgHnFCgsCLnetwavqgsp---------------ylCPqC ra
TRIM47: fs CpICl-epLre-----Pvt-LpCgHnFClaCLgalwphrgasgaggpgga-------arCPlC qe
TRIM32: le CpICm-esIteeqlr-Pkl-LhCgHtICrqCLekllassing---------------vrCPfC sk
TRIM41: av CaICl-dyFtd-----Pvs-IgCgHnFCrvCVtqlwggedeedrdeldre-/115/-rrCFtC pq
```

B

B-box1 DOMAIN

1 2 3 4 5 6 7 8

$CxxCx_{(7-15)}CxxCxxxxCxx[CH]x_{(3-4)}Hx_{(1-9)}H$

$CxxCx_{(3-11)}(ASV)xxxCxxCxxx(FLY)Cxx(CH)x_{(3-4)}Hx_{(1-9)}H$

```
 TRIM1: ia CqfCeqdpprd----AvktCitCevsYCdrC-ratHp-nkkpftsH rl
TRIM18: vl CqfCdqdpaqd----AvktCvtCevsYCdeClkatHp-nkkpftgH rl
 TRIM9: lk CqlCekapke-----AtvmCeqCDvfYCdpCrlrcHp-prgplakH rl
TRIM67: ai CqlCdrtppep----AatlCeqCdvlYCsaCqlkcHp-srgpfakH rl
TRIM36: im CdlCkpppqe-----StksCmdCsasYCneCfkihHp-wgtikaqH ey
TRIM46: il CqlCkppple-----AtkgCteCratFCneCfklfHp-wgtqkaqH ep
TRIM56: pa CalCplvggtstggpAtarCldCaddLCqaC-adgHr-ctrqtHtH rv
TRIM28: qc CtsCednap------AtsyCveCsepLCetC-veaHq-rvkytkdH tv
TRIM24: qv CtsCednae------AngfCveCvewLCktC-iraHq-rvkftkdH tv
TRIM33: qv CtsCednas------AvgfCveCgewLCktC-ieaHq-rvkftkdH li
TRIM66*:rn CseCkekra------AhilCtyCnrwLCssC-teeH—-r------H sp
TRIM45: lv CdlCndre-------VekrCqtCkanLChfC-cqaHr-rqkkttyH tm
TRIM71: hg CssCdegna------AssrCldCqehLCdnCv-raHq-rvrltkdH yi
TRIM44*:gt CdeCepdeapg----AeevCreCgfcYCrrH-aeaH--rqkflshH la
TRIM19: av CtrCkes--------AdfwCfeCeqlLCakC-feaHq-wfk-l--H ea
TRIM23: ir CdeDeahl-------AsvyCtvCathLCseCsqvtH--stktlakH rv
TRIM42: il CqvCrnrri------AykrCitCrlnLCndClkafH--sdvamqdH vf
 TRIM8: lh CvfCrrgpplp----AqkvClrCeapcCqsH-vqtHlqqpstargH ll
TRIM16*:vl CdfClddtrrvk---AvksCltCmvnYCeeH-lqpH-qvniklqsH ll
TRIM29*:vl CdsCignkqk-----AvksClvCqasFCelH-lkpHl-egaafrdH ql
TRIM25: va CdhClkea-------AvktClvCmasFCqeH-lqpHfd-spafqdH pl
TRIM47: vr CdaCpegaalp----AalsClsClasFCpaH-lgpHe-rspalrgH lv
```

C

B-box2 DOMAIN

1 2 3 4 5 6 7 8

$Cx_{(2-4)}\ Hx_{(6-9)}\ Cxx[CEHD]x_{(4-8)}\ CxxCx_{(3-6)}\ Hx_{(2-4)}\ [HC]$

$Cx_{(2-4)}Hx_{(3-6)}(FILMV)x_{(0-1)}(FY)x_{(0-1)}Cx_{(2)}[CDHE]x_{(3-7)}(ILMTV)Cx_{(2)}Cx_{(3-6)}Hx_{(2-4)}[HC]x_{(4)}(ILVMA)$

```
TRIM64: ni Cvl--Heetke-L---F-CeaDkr----lLCgpCsespe-Hma--H shspI
TRIM48*:qm Cgi--Hretkk-M---F-CevDrs----lLCllCsssqe-Hry--H rhcpA
TRIM49: qm Cgt--Hretkk-I---F-CevDrs----lLCllCsssqe-Hry--H rhrpI
TRIM43: qi Cgt--Hrqtkk-M---F-CdmDks----lLCllCsnsqe-Hga--H khhpI
 TRIM4: gl Cgr--Hwep---L-rlF-CedDqr----pVClvCresqe-Hqt--H amapI
TRIM39: sl Cpq--Hhea---L-slF-CyeDqe----aVCliCaisht-Hrp--H tvvpL
TRIM11: gv Cpa--Hrep---L-aaF-CgdElr----lLCaaCersge-Hwa--H rvrpL
TRIM52*:gm Cfk--Hqea---L-klF-CevDke----aICvvCresrs-Hkq--H svlpL
TRIM41: gi Cpk--Hqea---L-klF-CevDee----aICvvCresrs-Hkq--H svvpL
TRIM17: dl Cqe--Hhep---L-klF-CqkDqs----pICvvCresre-Hrl--H rvlpA
TRIM27: gv Cek--Hrep---L-klY-CeeDqm----pICvvCdrsre-Hrg--H svlpL
 TRIM7: ar Cgq--Hgep---F-klY-CqdDgr----aICvvCdrare-Hre--H avlpL
TRIM38: ms Cee--Hgeq---F-hlF-CedEgq----lICwrCerapq-Hkg--H ttalV
TRIM68: dl Cer--Hgek---L-kmF-CkeDvl----iMCeaCsqspe-Hea--H svvpM
TRIM58: rr Car--Hged---Lsr-F-CeeDea----aLCwvCdagpe-Hrt--H rtapL
TRIM21: er Cav--Hger---L-hlF-CekDgk----aLCwvCaqsrk-Hrd--H amvpL
TRIM22: dv Ceh--Hgkk---L-qiF-CkeDgk----vICwvCelsqe-Hqg--H qtfrI
TRIM34: dl Cdh--Hgek---L-llF-CkeDrk----vICwlCersqe-Hrg--H htvlt
 TRIM6: vl Cad--Hgek---L-qlF-CqeDgk----vICwlCersqe-Hrg--H htflV
 TRIM5: dh Car--Hgek---L-llF-CqeDgk----vICwlCersqe-Hrg--H htflt
TRIM31: at Cpr--Hqem---Fhy-F-CedDgk----fLCfvCreskd-Hks--H nvslI
TRIM26: kl Cer--Hrek---Lhy-Y-CedDgk----lLCvmCresre-Hrp--H tavlM
TRIM15: ty Cee--Hgek---I-yfF-CenDae----fLCvfCregpt-Hqa--H tvgfL
TRIM10: dv Cqe--Hgsrk--I-yfF-CedDem----qLCvvCreage-Hat--H tmrfL
TRIM40: yi Cpn--Hqkr---V-crF-Ce-Esrl---lLCveClvspe-Hms—-H heltL
TRIM50: kv Cvh--Hrnp---L-slF-CekDqe----lICglCgllgs-Hqh--H pvtpV
TRIM72: gh Cee--Hldp---L-siY-CeqDra----lVCgvCaslgs-Hrg--H rllpA
TRIM61: hv Ckk--Hnqv---L-tfF-CqkDle----lLCprCslstd-Hqh--H cvwpI
TRIM60: am Cek--Hnqf---L-tlF-CvkDle----iLCtqCsfstk-Hqk--H yicpI
TRIM75: tl Cek--Hnqp---L-svF-CkeDlm----vLCplCtqppd-Hqg--H hvrpI
TRIM20*:pq Ckr--Hlkqvq-L--lF-CedHde----pICliCslsqe-Hqg--H rvrpI
TRIM54: lm Cee--Heeek--I-niY-ClsCev----pTCslCkvfga-Hkd--C evapL
TRIM55: pm Cee--Heeer--I-niY-ClnCev----pTCslCkvfga-Hkd--C qvapL
TRIM63: pm Cke--Hedek--I-niY-CltCev----pTCsmCkvfgi-Hka--C evapL
TRIM13: pv Ckg--Hlgqp--L-niF-CltDmq----lICgiCatrge-Htk--H vfcsI
TRIM59: vt Cpe--Hyrqp--L-nvY-CllDkk----lVCghCltigq-Hhg--H piddL
 TRIM1: it Cld--Henek--V-nmY-CvsDdq----lICalCklvgr-Hrd--H qvasL
TRIM18: lm Cle--Hedek--V-nmY-CvtDdq----lICalCklvgr-Hrd--H qvaaL
 TRIM9: st Ctd--HelenhsM---Y-CvqCkm----pVCyqCleegk-Hss--H evkaL
TRIM67: pt Cpe--HemenysM---Y-CvsCrt----pVCylCleegr-Hak--H evkpL
TRIM36: lm Cpe--Heter--I-nmY-CelCrr----pVChlCklggn-Han--H rvttM
TRIM46: lm Cpd--Hkee---Vth-Y-CktCqr----lVCqlCrvrra-Hsg--H kitpV
 TRIM2: ls Cpn--Hdgnv--M-efY-CqsCet----aMCreCtege-Hae--H ptvpL
 TRIM3: fs Cpn--Hegkt--M-efY-CeaCet----aMCgeCrage-Hre--H gtvlL
TRIM56: aq Cpq--Hpgea--L-r-FlCqpCsq----lLCreCrldp--Hld--H pclpL
TRIM24: vf Cpf--Hkkeq--L-klY-CetCdk----lTCrdCqlle--Hke--H ryqfI
TRIM33: vf Cpv--Hkqeq--L-klF-CetCdr----lTCrdCqlle--Hke--H ryqfL
TRIM66*:ly Cpl--Htqev--L-klF-CetCdm----lTChsClvve--Hke--H rcrhV
TRIM28: vy Cnv--Hkhep--L-vlF-CesCdt----lTCrdCqlna--Hkd--H qyqfL
TRIM45: il Cpv--Hpaee--L-rlF-CefCdr----pVCqdCvvge--Hre--H pcdft
TRIM71: gf Cqh--Hddev--L-hlY-CdtCsv----pICreCtmgr--Hgg--H sfiyL
TRIM14*:wr Cpe--Hgdr---VaelF-CrrCrr----cVCalCpvlga-Hrg--H pvglA
TRIM44*:rk Cpd--Hgld---Lst-Y-CqeDrq----lICvlCpviga-Hqg--H qlstL
TRIM19: if CsnpnHrtpt--LtsiY-CrgCsk----pLCcsCalldssHselkC disae
TRIM37: dk Cen—-Hhek---Lsv-F-CwtCkk----cIChqCalwggmHgg--H tfkpL
TRIM23: tm Csq—-Hqvha--Ie--FvCleEgcqtsplMCcvCkeygk-Hqg--H khsvL
TRIM35: rv Crl--Hrgq---Lsl-F-CleDke----lLCcsCqadpr-Hqg--H rvqpV
TRIM42: ki Cih--Hpssr--Iie-Y-CrnDnk----lLCtfCkfsf--Hng--H dtisL
TRIM62: rp Cqa--Hdk----Vkl-F-CltDra----lLCffCdepal-Heq--H qvtgI
 TRIM8: ws Cpq--Hnayr--L---YhCeaEqv----aVCqyCcyysgaHqg--H svcdV
TRIM16*:ry Cpa--Hhsp---Lsa-F-CcpDqq----cICqdCcqe---Hsg--H tivsL
TRIM29*:rk Cpv--Hgkt---Mel-F-CqtDqt----cICylCmfqe--Hkn--H stvtV
TRIM25: rk Csq--Hnrlre-F---F-CpeHse----cIChiClve---Hkt--C spasL
TRIM47: sl Cpr--Hlrp---Ler-Y-CraErv----cLCeaCaaqe--Hrg--H elvpL
TRIM65: ar Cpr--Hgrp---Lel-F-CrtEgr----cVCsvCtvre--crl--H erallL
TRIM32: lm Crs--Cgrr---LprqF-CrsCgl----vLCepCread--HqppgH ctlpV
```

Additional file 1. **A**) Alignment of the RING domains of 61 out of the 68 TRIM proteins. TRIM14, 16, 20, 29, 44, 66 are not included because they lack this domain. TRIM74 is not shown because its RING domain is identical to that of TRIM73; Two consensi are shown: at the top is the general consensus for the TRIM RING finger, the conserved cysteine (C) and histidine (H) are in bold and numbered, x can be any amino acids, when these residues are more than two, the numbers are subscript and indicated in parentheses; at the bottom is the detailed consensus showing in capital letters the residues preferentially found in that position (>95%). The region from -2 with respect to the first C and +2 with respect to the last C has been used to produce the alignment. The conserved C and H are shaded and the other multiple-choice conserved residues are in capital letters, exceptions to these conserved residues are in bold small letters. **B**) Alignment of the B-box1 domain of 22 TRIM proteins. The legend is as in A). **C**) Alignment of the B-box2 domain of 66 TRIM proteins. TRIM73 and 74 are not shown because their B-box2 domain is identical to that of TRIM50. The legend is as in A).
