# Supplementary material for: Genomic analysis of the TRIM family reveals two groups of genes with distinct evolutionary properties
Source: BMC Evol Biol. 2008 Aug 1;8:225. doi: 10.1186/1471-2148-8-225 (PMC2533329; doi:10.1186/1471-2148-8-225)
Supplement: Additional file 2 — Reports the values of Coiled-coil predictions for all the human TRIM and TRIM-like proteins. [file 1471-2148-8-225-S2.pdf]

Additional file 2. TRIM proteins Coiled-coil prediction

	**Coiled-coil score[a] (aa[b])**	
	**window=28[c]**	**window=21**
TRIM1	**1** (0-60); **0.5** (80-115)	**1** (0-60)
TRIM2		**0.6** (5-35)
TRIM3	**0.78** (10-40); **1** (40-80)	**0.9** (10-35); **0.95** (40-70)
TRIM4	**1** (65-110)	**0.95** (65-105)
TRIM5	**1** (0-50); **1** (70-110)	**1** (5-45); **1** (70-120)
TRIM6	**1** (5-50); **1** (70-120)	**1** (5-45); **1** (70-120)
TRIM7	**0.9** (5-95)	**1** (5-35); **0.7** (40-60); **0.95** (65-110)
TRIM8	**1** (0-90)	**1** (0-90)
TRIM9	**1** (10-55)	**0.9** (10-50); **0.6** (60-90)
TRIM10	***0.9** (15-50)[d]*	***0.5** (15-45)[d]*
TRIM11	**1** (5-40); **1** (45-90)	**1** (5-35); **1** (45-90)
TRIM13	***0.75** (45-80)[d]*	***0.7** (50-80)[d]*
TRIM14*	**0.95** (20-50)	**0.7** (25-50)
TRIM15	**1** (40-80)	**1** (15-35); **1** (45-85); **1** (90-120)
TRIM16*	**0.9** (5-45); **0.9** (80-110)	**0.9** (5-45)
TRIM17	**0.85** (15-50); **0.8** (70-120)	
TRIM18	**1** (5-60)	**1** (10-45); **0.5** (45-70)
TRIM19	**0.5** (70-100)	
TRIM20*		**0.9** (5-35)
TRIM21	**1** (0-45); **1** (60-110)	**1** (5-45); **1** (60-120)
TRIM22	**1** (5-50); **1** (65-120)	**1** (10-50); **0.8** (70-90); **0.9** (90-120)
TRIM23	**0.85** (130-175)	**0.95** (135-170)
TRIM24	**1** (60-110)	**0.95** (70-110)
TRIM25	**1** (30-80); **1** (90-130)	**1** (40-75); **1** (90-130)
TRIM26	**1** (55-95)	**1** (60-95)
TRIM27	**1** (5-50)	**0.95** (5-40)
TRIM28		**0.7** (0-25)
TRIM29*	**1** (5-100)	**1** (5-50); **1**(50-100)
TRIM31	**0.95** (0-40); **1** (145-180)	**0.95** (5-35); **0.8** (145-170)
TRIM32	**1** (5-60)	**1** (5-55)
TRIM33	**0.95** (0-30); **0.9** (35-70)	**0.9** (0-30); **1** (35-65)
TRIM34	**1** (5-50); **1** (60-115)	**1** (5-50); **1** (60-115)
TRIM35	**1** (80-120)	**1** (85-120)
TRIM36	***0.95** (30-60)[d]*	***0.85** (35-60); **0.9** (80-100)[d]*
TRIM37	**1** (5-60); **0.95** (65-110)	**1** (5-60); **0.95** (80-110)
TRIM38	**1** (5-40)	**1** (5-40); **0.8** (100-120)
TRIM39	**1** (40-110)	**1** (45-110)
TRIM40	**1** (5-55)	**1** (10-50); **0.7** (60-90)
TRIM41	**1** (20-60); **0.95** (80-120)	**0.95** (25-60); **1** (90-120)
TRIM42		**0.9** (60-90)
TRIM43	**0.8** (5-40)	**1** (65-95)
TRIM44*	**1** (80-115)	**1** (85-115)
TRIM45	**0.7** (25-55); **1** (60-120)	**1** (60-115)
TRIM46	**0.95** (65-100)	**1** (60-100); **0.85** (120-145)
TRIM47	**0.8** (80-110)	**1** (85-110)
TRIM48*	*short sequence after the B-box*	
TRIM49	***0.55** (90-115)[e]*	***0.6** (90-115)[e]*
TRIM50	**1** (5-50); **0.8** (80-115)	**1** (5-50); **0.8** (80-115)
TRIM52*	*short sequence after the B-box*	
TRIM54	**1** (100-150)	**0.95** (105-145)
TRIM55	**0.95** (55-100)	**0.95** (55-100)
TRIM56	**1** (20-60); **1** (65-115)	**1** (25-60); **0.95** (65-115)
TRIM58	**1** (65-120)	**0.75** (15-40); **1** (65-120)
TRIM59		**0.85** (30-55); **0.7** (100-120)
TRIM60	**0.95** (85-120)	
TRIM61	***0.9** (45-90)[d]*	***0.95** (45-80)[d]*
TRIM62	**1** (0-60); **1** (70-120)	**1** (5-50); **1** (80-120)
TRIM63	**1** (70-120)	**1** (80-120)
TRIM64	**1** (60-110)	**1** (60-100)
TRIM65	**1** (10-50); **0.7** (85-120)	**0.95** (10-45)
TRIM66*	**0.95** (25-50); **1** (50-100)	**0.8** (30-50); **1** (50-100)
TRIM67	**0.95** (15-55)	**0.9** (30-50)
TRIM68	**0.7** (80-120)	**0.75** (20-40); **1** (80-110)
TRIM71	**0.9** (25-75); **1** (80-120)	**1** (80-120)
TRIM72	**0.9** (10-50); **1** (85-115)	**0.8** (20-45); **1** (90-115)
TRIM73	**1** (5-50); **0.85** (85-115)	**1** (5-45); **0.85** (85-115)
TRIM74	**1** (5-50); **0.85** (85-115)	**1** (5-45); **0.85** (85-115)
TRIM75	**0.8** (40-80)	**0.7** (40-80)

[a] Coils score (probability) (http://www.ch.embnet.org/software/COILS_form.html)

[b] numbering starting from the last aa of the B-box2 domain (aa no. 1)

[c] Scores >0.5 are shown

[d] The score obtained with only the unweighted option

[e] The score obtained with only the weighted option
