# Supplementary material for: Genomic analysis of the TRIM family reveals two groups of genes with distinct evolutionary properties
Source: BMC Evol Biol. 2008 Aug 1;8:225. doi: 10.1186/1471-2148-8-225 (PMC2533329; doi:10.1186/1471-2148-8-225)
Supplement: Additional file 3 — Shows the unrooted phylogenetic trees generated from the alignments of single domains of the tripartite motif. [file 1471-2148-8-225-S3.pdf]

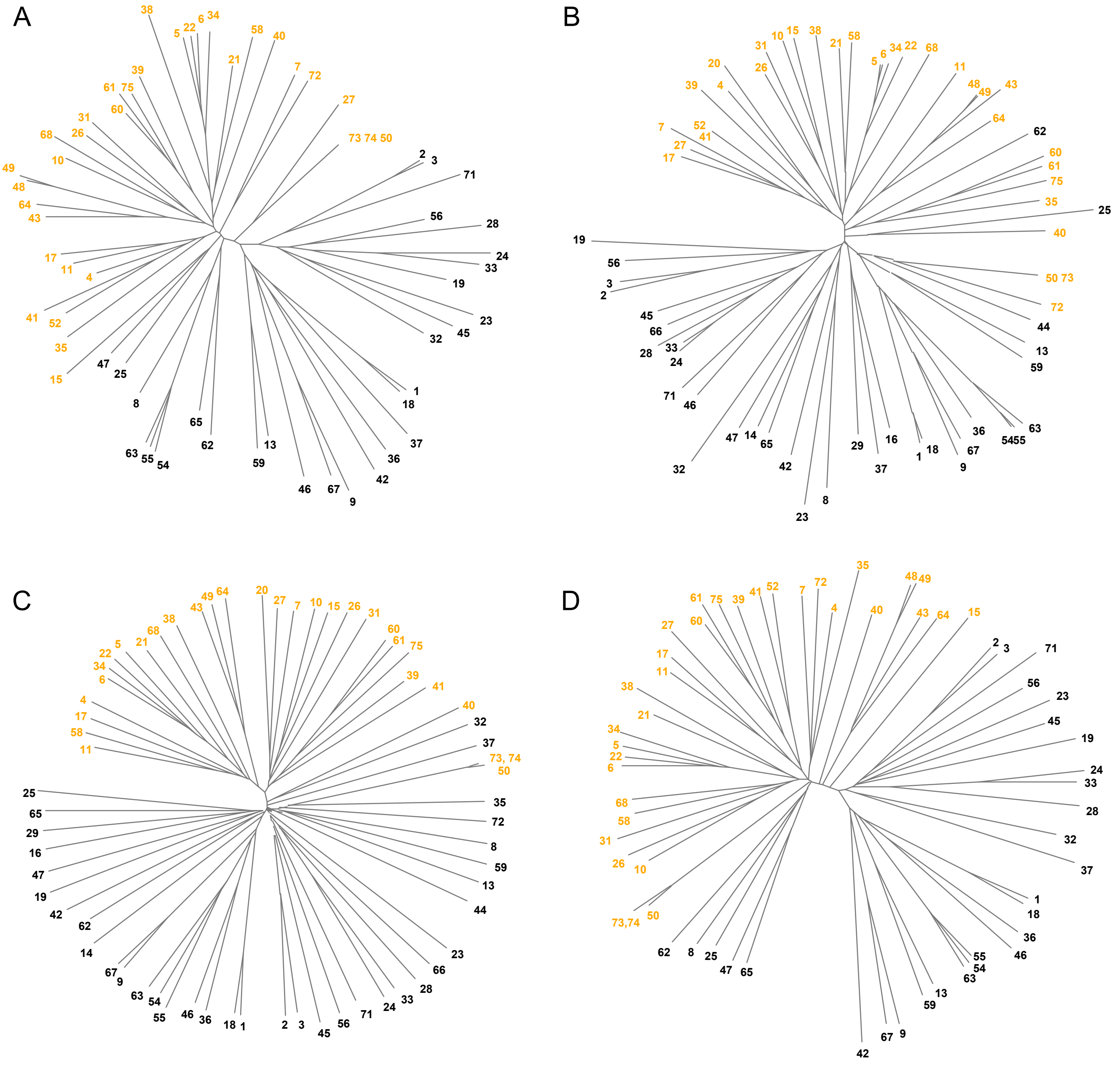

Additional file 3. Unrooted phylogenetic trees generated from the alignments of A) RING finger; B) B-box2; C) Coiled-coil region; D) RING finger and the spacer between RING and the first B-box present. Numbers indicate TRIM family members; in ochre are the Group 2 and in black the Group 1 TRIM genes.
