# Supplementary material for: Genomic analysis of the TRIM family reveals two groups of genes with distinct evolutionary properties
Source: BMC Evol Biol. 2008 Aug 1;8:225. doi: 10.1186/1471-2148-8-225 (PMC2533329; doi:10.1186/1471-2148-8-225)
Supplement: Additional file 4 — Shows a schematic representation of the human TRIM genomic clusters. [file 1471-2148-8-225-S4.pdf]

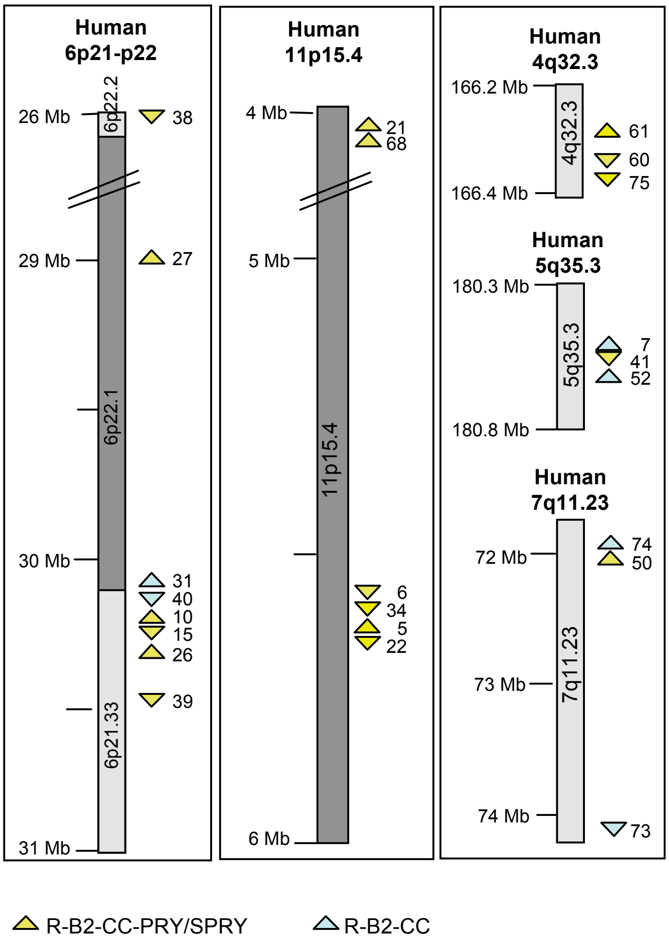

Additional file 4. Schematic representation of the TRIM genes located in clusters on human chromosome 6p21-22, 11p15.4, 4q32.3, 5q35.3, and 7q11.23. Six of the 8 TRIM genes present at 6p21.33-p22.1 are comprised in approximately 500 kbp; six genes, which lie in less than 2 Mbp at 11p15.4, include a core of 4 genes within approximately 300 kbp; the other human clusters, composed of three TRIM genes each, are located at 4q32.3, 5q35.3, and 7q11.23 The TRIM genes are indicated as arrowheads and numbered; the arrowhead are oriented according to the direction of transcription. The color of the arrowhead indicates the domain composition of the TRIM genes as described at the bottom of the figure.
