# Supplementary material for: Genomic analysis of the TRIM family reveals two groups of genes with distinct evolutionary properties
Source: BMC Evol Biol. 2008 Aug 1;8:225. doi: 10.1186/1471-2148-8-225 (PMC2533329; doi:10.1186/1471-2148-8-225)
Supplement: Additional file 5 — Shows comparative and evolutionary analyses of the cluster of TRIM5, 6, 22, and 34 in mammals. [file 1471-2148-8-225-S5.pdf]

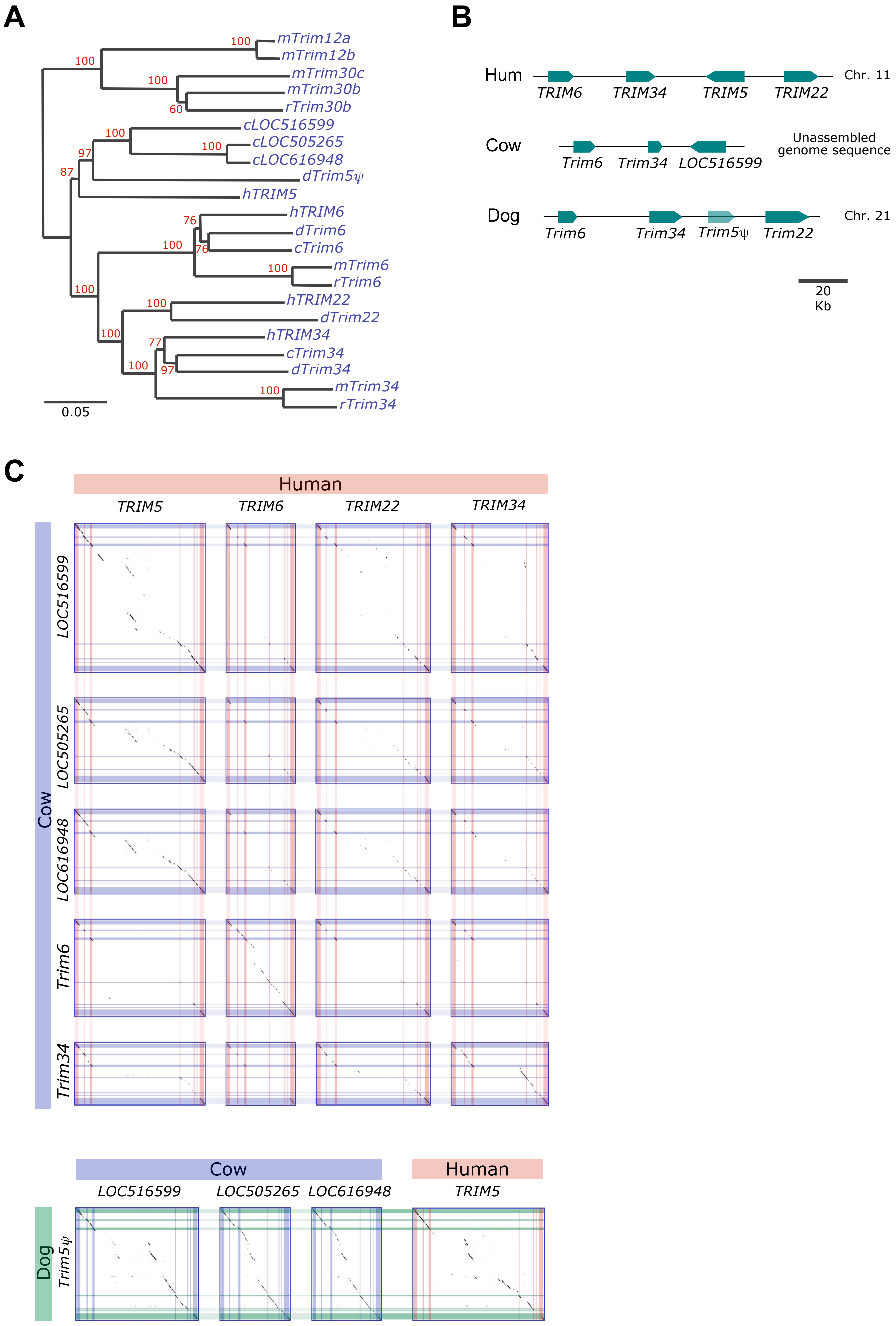
A
mTrim12a
mTrim12b
mTrim30c
mTrim30b
rTrim30b
cLOC516599
cLOC505265
cLOC616948
dTrim5ψ
hTRIM5
hTRIM6
dTrim6
cTrim6
mTrim6
rTrim6
hTRIM22
dTrim22
hTRIM34
cTrim34
dTrim34
mTrim34
rTrim34
0.05
B
Hum
TRIM6
TRIM34
TRIM5
TRIM22
Chr. 11
Cow
Trim6
Trim34
LOC516599
Unassembled genome sequence
Dog
Trim6
Trim34
Trim5ψ
Trim22
Chr. 21
20 Kb
C
Human
TRIM5
TRIM6
TRIM22
TRIM34
Cow
LOC516599
LOC505265
LOC616948
Trim6
Trim34
Cow
Human
LOC516599
LOC505265
LOC616948
TRIM5
Dog
Trim5ψ

Additional file 5. Genomic and evolutionary analysis of *TRIM5*, *6*, *22*, *34*. (**A**) Phylogenetic analysis of human (h) coding sequences of *TRIM5*, *6*, *22*, *34* and their homologous sequences from mouse (m), rat (r), cow (c) and dog (d). Bootstrap support values above 50% based on 1000 replicates are shown above the nodes. Evolutionary distances are represented as nucleotide substitutions by the scale bar. *TRIM6* and *34* orthologs are present in all organisms, whereas a *TRIM22* ortholog is present only in dog. *TRIM5* segregates with a dog pseudogene we named *Trim5ψ* and with cow *LOC516599*, *LOC616948*, and *LOC505265*. TRIM genes of groups 12 and 30 are only present in rodents and form a separate clade, as previously shown (Asaoka *et al.*, BBRC 2005, 338:1950-1956; Song *et al.*, J Virol 2005, 79(10):6111-6121). This topology was supported by several different tree-building methods (see Materials and Methods for details) and suggests that human *TRIM5*, dog *Trim5ψ* and cow *LOC516599*, *LOC616948*, and *LOC505265* were derived from the same ancestor gene after separation from the ancestors of *TRIM6*, *22*, and *34*. (**B**) Genomic organization of the loci encoding *TRIM5*, *6*, *22*, *34*, and related sequences in humans, cow, and dog. Genes (green head arrows) are oriented according to the direction of transcription. The entire dog locus could be superimposed to the human counterpart, with the only difference of the inversion of the *TRIM5* ortholog *Trim5ψ*. A complete representation of the cow locus was not possible, due to the lack of a wider assembling of the concomitant genomic sequences. Therefore, the position of cow *LOC616948* and *LOC505265* relatively to the genomic locus including *LOC516599* could not be determined. (**C**) Dot-plot analysis of human *TRIM5*, *6*, *22*, *34* genes, dog *Trim5y* pseudogene, and their cow counterparts. In each single diagram, the genomic sequences of the given gene pair are compared; a dot is inserted when the regions at its *x* (first gene) and *y* (second gene) coordinates share an established degree of sequence similarity (see Materials and Methods for details). In each diagram, genes are oriented with their 5' at the left in the *x*-axis and at the top in the *y*-axis. Red, cyan, and green bars represent the exons of the human, cow, and dog genes, respectively. Exonic sequence similarity is observed for all gene pairs. As expected, intronic sequence similarity is significant for the orthologous pairs (*hTRIM6*/*cTrim6*, *hTRIM34*/*cTRIM34*, *hTRIM5*/*dTrim5ψ*) but undetectable for the obvious paralogous pairs (*hTRIM5*/*cTrim6*, *hTRIM6*/*cTRIM34* etc.). Both human *TRIM5* and dog *Trim5ψ* show extensive intronic sequence similarity to cow *LOC516599*, *LOC616948*, and *LOC505265*. Conversely, these cow loci do not show intronic similarity to any of the other examined genes. Taken together, these analyses strongly suggested that a locus including *TRIM5*, *6*, *22*, and *34* was present in the last common ancestor of humans, cow, and dog, differently from what previously reported (Si *et al.*, PNAS 2006, 103(19): 7454-7459). While *TRIM6* and *34* orthologs have been conserved in all organisms, *TRIM22* has been retained only in dog among the species examined. *TRIM5* has an orthologous pseudogene in dog, and both shares locus similarity with cow *LOC516599*, *LOC616948*, and *LOC505265*, which are clearly paralogs derived by locus duplication of a common ancestor (possibly the *TRIM5* ancestor) after cow-dog lineage split. Therefore, this analysis supports a common origin for *TRIM5* and its functional ortholog *LOC516599*. Dot-plot analysis was performed at http://www.vivo.colostate.edu/molkit/dnadot by combining the following pairs of window/stringency values: 49/17, 99/45, 199/105, and 399/239.
